# Iron overload suppresses LKB1 and induces IL36G anti-tumor immunity in PDAC metastasis

**DOI:** 10.1126/sciadv.adz8681

**Published:** 2026-07-17

**Authors:** Douglas E. Biancur, Harsha Venkatesh, Amy Crawford, Yealeen Jeong, Albert S.W. Sohn, Kevin S. Kapner, Keisuke Yamamoto, Elaine Y. Lin, Robert S. Banh, Mohamad Assi, Beny Shapiro, Peter Yu, Soomin C. Song, William A. Coetzee, Andrew J. Aguirre, Alisha N. Jones, Alec C. Kimmelman, Richard Possemato

**Affiliations:** ^1^ Department of Pathology, New York University Grossman School of Medicine; New York, NY 10016 USA.; ^2^ Perlmutter Cancer Center, New York University Grossman School of Medicine; New York, NY 10016, USA.; ^3^ Department of Chemistry, New York University, New York, NY 10003, USA.; ^4^ Department of Radiation Oncology. New York University Medical Center; New York, NY 10016, USA.; ^5^ Department of Medical Oncology, Dana-Farber Cancer Institute, Boston, MA 02215, USA.; ^6^ Broad Institute of MIT and Harvard, Cambridge, MA 02142, USA.; ^7^ Department of Gastroenterology, Graduate School of Medicine, The University of Tokyo, Tokyo, Japan.; ^8^ Department of Biochemistry and Molecular Pharmacology, New York University School of Medicine; New York, New York 10016, USA.; ^9^ Division of Hematology & Medical Oncology, New York University Medical Center; New York, NY 10016 USA.; ^10^ Department of Neuroscience & Physiology, NYU Grossman School of Medicine, New York, NY 10016 USA.

## Abstract

Pancreatic ductal adenocarcinoma (PDA) is an aggressive cancer that frequently presents with disseminated disease. The PDA metastatic microenvironment imposes distinct metabolic stressors, potentially generating context-dependent vulnerabilities. Therefore, we employed CRISPR-based genetic screening in a model of PDA liver metastasis to identify novel and possibly targetable liabilities. Remarkably, ferritin heavy chain (FTH1) emerged as the most prominent liver-specific dependency – loss of FTH1 suppressed tumor growth specifically in the liver microenvironment. FTH1 deletion and subsequent disruption of iron handling triggers mitochondrial dysfunction and ionic imbalance, including cytosolic calcium overload. These perturbations result in the activation of a transcriptional program that triggers anti-tumor immunity mediated by immunostimulatory cytokine IL36G. Mechanistically, FTH1 deletion and subsequent ionic imbalance causes decreased protein levels of the tumor suppressor *Stk11* (LKB1) which we propose to be mediated by an RNA G-quadruplex located in the 5′-UTR of LKB1. The loss of LKB1 protein levels alters signaling cascades resulting in reduced SIK signaling and inhibition of nonsense mediated decay, ultimately leading to *Il36g* mRNA stabilization. Taken together, this work elucidates novel ionic disruptions that regulate the translation of LKB1 through a previously undescribed quadruplex in the 5′UTR, altering signaling axes that can be targeted to generate an anti-tumor immune response in PDA.

## Teaser

Ion imbalance stabilizes an RNA G-quadruplex in LKB1 disrupting downstream signaling cascades driving anti-tumor immunity.

## INTRODUCTION

Cancer metastasis remains the leading cause of cancer-related mortality, with metastatic pancreatic cancer representing a particularly devastating example (*1*–*3*). Indeed, over 50% of patients diagnosed with pancreatic cancer have metastases and the liver is the most common site of disseminated disease (*4*). These data underscore the critical need to understand the mechanisms that drive metastatic cancer cell progression and survival to better combat this disease.

To successfully metastasize to the liver, pancreatic cancer cells must adapt their metabolic networks to support growth in the distinct conditions they encounter both during the metastatic cascade and in the new environment in which they colonize. While in vitro models have been instrumental in unraveling the intricacies of cancer cell biology, they inherently overlook the dynamic and multifaceted microenvironments that cancer cells encounter in vivo. For example, cancer cells must survive growth in detached conditions.

Here, we use genetic screening with a metabolism-focused library to define the metabolic requirements for pancreatic cancer liver metastasis. We find that proper iron storage is important for liver metastatic growth and that iron overload can result in metabolic and signaling defects that depress translation of the LKB1 tumor suppressor, dependent on regulation within the LKB1 5′UTR. Reduced LKB1 expression and alterations in downstream signaling in turn leads to activation of the inflammatory cytokine IL36G and enhanced tumor control. We propose that this regulation of LKB1 may be a common stress response to ionic dyshomeostasis that, when triggered, can promote immune-mediated tumor control.

## RESULTS

### FTH1 knockout is a liver-specific metabolic liability that acts via both cell autonomous and non-cell autonomous mechanisms

**Fig. 1.**
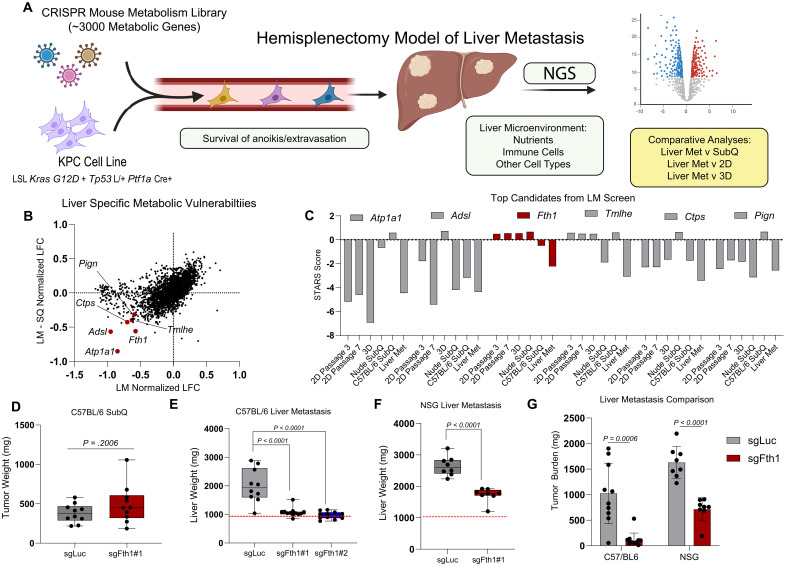
Fth1 KO is alLiver specific metabolic liability that acts via both cell-autonomous and Nnn-cell-autonomous mechanisms. **A**) Schematic of vivo metabolism-focused CRISPR/Cas9 metastasis screen, syngeneic KPC cell line created in BioRender. Biancur, D. (2026) https://BioRender.com/768288i. (**B**) Identification of liver-specific metabolic genetic dropouts comparing metastasis screen to published data of subcutaneous tumors. (LM, liver metastases; SQ, C57BL/6 subcutaneous screen; LFC, Log^2^ Fold-change comparing LM screen to SQ screen). (**C**) STARS scores depicting significantly depleted genes (FDR < 0.2) in metastasis screen and other screening conditions. (**D**) Implantation of *sgLuc* (*n* = 10) and *sgFth1* (*n* = 10) cells, syngeneic subcutaneous tumors. Data are median ± range of individual tumor weights. (**E**) Implantation of *sgLuc* (*n* = 10) and *sgFth1* (*sgFth1#1 n* = 10, *sgFth1#2 n* = 12) cells into the liver, syngeneic C57BL/6 liver metastases. Data are median ± range of individual tumor weights. Red line represents sham injection liver weight. (**F**) Implantation of *sgLuc* (*n* = 8) and *sgFth1* (*n* = 8) cells in NOD.Cg-*Prkdc^scid^ Il2rg^tm1Wjl^*/SzJ (NSG) liver metastases. Data are median ± range of individual tumor weights. Red dotted line represents sham injection liver weight. (**G**) Comparison of panels (E) and (F) plotting tumor burden (total liver weight – sham injection liver weight) between immunocompetent (C57BL/6) and immunocompromised hosts (NSG). Statistical analysis performed using GraphPad Prism. Unpaired, two tailed Student’s t-tests performed when comparing two groups.

To elucidate the metabolic dependencies of pancreatic cancer cells that specifically metastasize to the liver, we conducted an in vivo CRISPR-Cas9 screen in a cell line isolated from a genetically engineered mouse model of PDA harboring oncogenic Kras and loss-of-function p53 mutations (KPC), utilizing the hemisplenectomy model of liver metastasis and a mouse metabolism sgRNA library which targets a comprehensive set of ~2,900 metabolic genes (Fig. 1A, Supplementary Table). We compared the findings from the hemisplenectomy screen to our previously published CRISPR screens using the same cell line (HY19636) and sgRNA library performed both in vitro (2D and 3D culture) and in vivo (subcutaneous tumors) (*5*). By plotting the differential dependencies between the liver metastasis screen and previously published datasets we uncovered liver metastasis specific metabolic liabilities (Fig. 1, B and C). The vast majority of genetic dependencies were highly conserved and represented in both the subcutaneous and liver metastasis models exemplifying the robustness of our in vivo metastasis platform (Fig. 1B). There were, however, some sgRNAs depleted specifically in the context of the liver metastasis screen thus suggesting the possibility that these sgRNAs target genes exhibiting tissue or metastasis-specific metabolic liabilities. When comparing significant dependencies across these screens, ferritin heavy chain (FTH1) emerged as the most representative liver specific vulnerability, as it was selectively important for cellular fitness in liver metastases (Fig. 1C). Along with ferritin light chain (FTL), FTH1 is a component of the iron storage complex, ferritin. FTH1 has oxidoreductase activity that enables storage of ferric iron (Fe^3+^) and limits the levels of highly reactive ferrous iron (Fe^2+^) (*6*). We validated our findings by generating control (*sgLuc*) and *Fth1* knockout (*sgFth1*) cell lines using two independent sgRNAs and assaying the cells across the different screening conditions (fig. S1A). Indeed, *sgFth1* cells exhibited no growth phenotype when cultured in the 2D or 3D setting as predicted by our screening data (fig. S1, B and C). Furthermore, when *sgFth1* cells were implanted subcutaneously into either C57BL/6 or Athymic Nude mouse models, we also observed no differences in tumor size (Fig. 1D and fig. S1D). Additionally, to determine if FTH1 loss resulted in growth suppression in the pancreatic microenvironment, we injected tumor cells orthotopically into the pancreas of C57BL/6 hosts, which revealed no change in tumor growth between *sgLuc* and *sgFth1* cell lines (fig. S1E). Finally, we used the hemisplenectomy liver metastasis model and observed a marked reduction in metastatic tumor burden in the *sgFth1* condition when compared to controls, validating the results of our screen (Fig. 1E). Taken together, these data support the hypothesis that FTH1 is critical for pancreatic cancer growth and fitness specifically in the context of liver metastasis. We then validated key experiments in another syngeneic KPC pancreatic cancer cell line (HY15549) and demonstrated no growth suppression in 2D culture but a marked growth disadvantage in the hemisplenectomy model (fig. S1, F to H).

While these data imply that FTH1 is necessary for liver metastasis, we explored two potential explanations for the reduced tumor burden upon knockout (KO): I) Functional FTH1 is critical for the metastatic cascade or survival of anoikis, and II) FTH1 is important for outgrowth in the liver. To differentiate between these two possibilities, we employed a metastasis model directed toward the lung, another common site for pancreatic cancer metastasis. We discovered that *sgFth1* cells were able to colonize and grow in the lung at similar rates as controls (fig. S1I). These data suggest that the decreased tumor burden observed in the hemisplenectomy model was due to the liver microenvironment, implying that loss of FTH1 is a tissue specific metabolic liability. To test this, we performed a direct injection of control and *sgFth1* cells into the liver, thereby bypassing the metastatic process. This experiment revealed a decrease in tumor burden in FTH1 KO conditions when compared to controls, indicating that FTH1 is required for tumor formation in the liver microenvironment (fig. S1J). Since these experiments were performed in syngeneic hosts with intact immune systems, we wanted to understand if loss of FTH1 was acting in a cancer cell autonomous manner or if loss of FTH1 was triggering an immune response. To test this hypothesis, we assessed liver metastasis using the hemisplenectomy model in NSG mice, which lack a functional immune system (Fig. 1F). By plotting the metastatic tumor burden in the immunocompetent versus immunocompromised models, we could demonstrate that there was an approximately a two-fold difference in tumor burden between the control and *sgFth1* cells in the NSG background versus an approximately eight-fold difference in that of the C57BL/6 background (Fig. 1G). These data suggest that FTH1 loss is acting in both a cell-autonomous and non-cell-autonomous manner to impair tumor growth in the liver microenvironment. We reasoned that understanding the importance of how FTH1 promotes cell growth and protects cells from immune surveillance in the liver microenvironment may help us better understand more about pancreatic cancer liver metastasis and design better therapy.

### Iron overload causes mitochondrial dysfunction and glycolytic rewiring in ferritin deficient cells

**Fig. 2.**
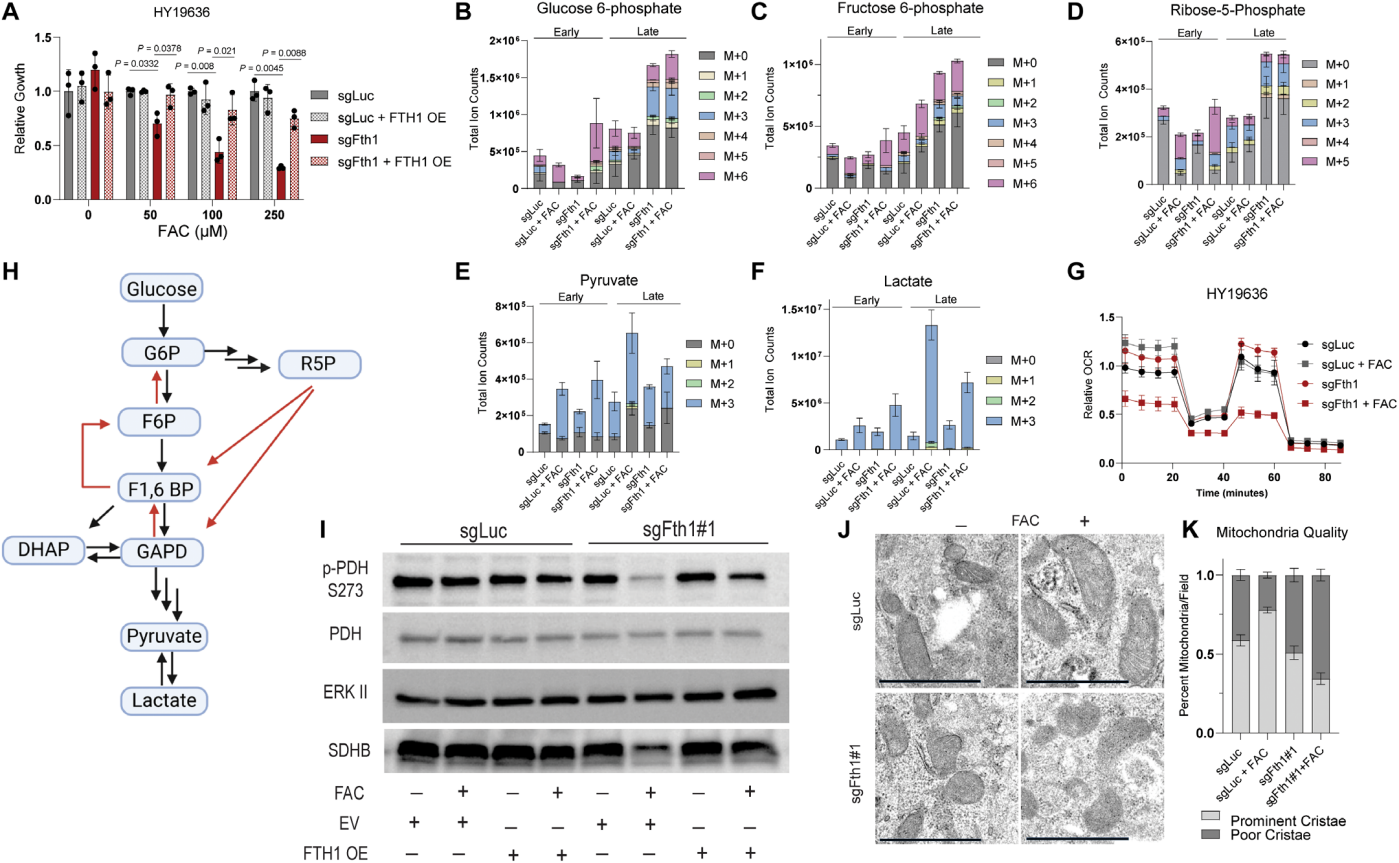
Iron overload causes mitochondrial dysfunction and glycolytic rewiring in ferritin deficient cells. (**A**) Relative proliferation rates of *sgLuc* and *sgFth1* cells in untreated or FAC treated conditions at concentrations described. Data are relative cell proliferation in arbitrary units. Data are mean ± SD of three independent wells, representative experiment. (**B** to **F**) Total ion current and labeling pattern of indicated metabolites from U-^13^C-glucose tracing, 20 minutes, *sgLuc* and *sgFth1* cells treated with FAC (24 hours or 56 hours). Data are mean ± SD of three independent wells, representative experiment. (**G**) Relative OCR of *sgLuc* and *sgFth1* cells untreated or treated with FAC. Data are plotted as relative OCR to untreated *sgLuc* samples. Data presented as mean and error bars depict ± SD of six individual wells from a representative experiment. (**H**) Schematic of glucose carbon utilization in cells depicting glucose carbon shunting into the oxidative arm of the pentose phosphate pathway (PPP) in *sgFth1* cells at late time points. The distinct M + 3 labeling patterns is a result of glucose carbon reversing in upper glycolysis likely as a means of increasing cycling through the PPP. (**I**) Representative immunoblot of *sgLuc* and *sgFth1* cells infected with either empty vector (EV) or *Fth1* cDNA (FTH1 OE) that were untreated or treated with FAC for 48 hours and probed for phospho-PDH S273, total PDH, ERK II, and SDHB. (**J**) Representative electron microscopy images of mitochondria in either *sgLuc* or *sgFth1* cells untreated or treated with FAC. Scale bar represents 1um. (**K**) Quantification of mitochondrial quality from electron microscopy images assessing cristae and intermembrane space architecture. Assessment was performed in a blinded manner on 10 EM images per condition. Data are mean ± SEM. Statistical analysis was performed using GraphPad Prism. Unpaired, two tailed Student’s t-tests were performed when comparing two groups to each other.

Since loss of FTH1 specifically impairs tumor growth in the liver, we considered unique metabolic features of the liver microenvironment that might explain this observation. The liver is a critical site for iron regulation and metabolism that is reported to be particularly high in iron compared to other tissues (*7*). Indeed, ICP-MS confirmed that the saline-perfused liver contains higher iron content than the pancreas and lung (fig. S2, A and B). Liver metastasis has been shown to induce tissue damage as measured by functional assays, which may additionally release iron from intracellular stores (*8*). We reasoned that loss of FTH1 and an inability to sequester ferrous iron in this environment may result in iron overload and have an impact on tumorigenesis. Iron overload is implicated in pathophysiology, and high cytosolic ferrous iron can catalyze membrane lipid oxidation, triggering a non-apoptotic form of cell death termed ferroptosis (*9*, *10*). To understand if high iron levels could impact *sgFth1* cell proliferation, we modeled a high iron environment in cell culture using ferric ammonium citrate (FAC) as our iron source. We observed a dose-dependent inhibition of cancer cell proliferation that could be rescued by overexpression of a guide-resistant *Fth1* cDNA (Fig. 2A and fig. S2C). No cell death was observed and treatment with the ferroptosis suppressor Ferrostatin was unable to support growth, demonstrating that the reduced growth was not due to ferroptosis (fig. S2, D and E). While RNA sequencing on control and *sgFth1* cells treated with FAC revealed a striking upregulation of antioxidant response genes upon FTH1 loss that could be rescued upon antioxidant treatment (fig. S2, F to H), antioxidant treatment was unable to restore the growth of *sgFth1* cells under high iron conditions (fig. S2, I and J) thus demonstrating that REDOX imbalance was not the underlying mechanism of reduced cellular proliferation or triggering ferroptosis.

Considering iron is an essential element for a multitude of metabolic processes, we performed U-^13^C-glucose tracing to understand how glucose carbon utilization was being impacted by high iron conditions (*6*). We observed a number of time-dependent changes to glucose carbon utilization (Fig. 2, B to F and fig. S2, K to L). Stable isotope tracing with uniformly labeled glucose revealed unexpected isotopologue distributions in upper glycolytic and pentose phosphate pathway (PPP) intermediates. In addition to increased total pool sizes at late timepoints, glucose-6-phosphate (G6P) and fructose-6-phosphate (F6P) exhibited a pronounced enrichment of M + 3 isotopologues, which is inconsistent with simple forward glycolytic flux from U-^13^C_6_ glucose (Fig. 2, B and D and fig. S2, K to O). As these metabolites are typically fully labeled by glucose, an M + 3 labeling pattern is consistent with excessive ROS generated by free iron activating a program to recycle glucose-derived carbon in upper glycolysis via the oxidative arm of the PPP, likely through oxidation of the GAPDH redox switch (Fig. 2H) (*11*, *12*). Ribose-5-phosphate (R5P) levels were modestly increased, however, its isotopologue distribution remained comparatively mixed and did not mirror the M + 3 enrichment observed in hexose phosphates (Fig. 2C and fig. S2M). These data suggest that although glucose-derived carbon enters the PPP, ribose production is not the dominant metabolic fate. Collectively, these data support a model in which the PPP functions primarily as a carbon-recycling and metabolic buffering pathway to support NADPH synthesis under iron induced oxidative stress.

Notably, we also observed pyruvate and lactate accumulate in both *sgLuc* and *sgFth1* cells upon extended iron overload (Fig. 2, E and F and fig. S2, N and O). This finding is consistent with our observation of FAC-induced transcriptional downregulation and decreased protein abundance of the monocarboxylate transporter SLC16A3, which exports lactate produced by glycolysis (fig. S3, A and B).

We hypothesized that under iron overload and rewired upper glycolysis, cells will rely more on mitochondrial metabolism to meet their anabolic needs. Recently, it has been demonstrated that lactate accumulation results in the upregulation of oxidative phosphorylation (OXPHOS), thereby acting as a mitochondrial messenger (*13*). Therefore, we measured the oxygen consumption rate (OCR) and TCA cycle metabolites of *sgFth1* and *sgLuc* cells treated with FAC (Fig. 2G and fig. S3, C to R). In control cells, we observed increased OCR in FAC-treated conditions, consistent with lactate accumulation stimulating mitochondrial OXPHOS. In contrast, *sgFth1* cells failed to respond to FAC-induced lactate accumulation and instead exhibited a reduced OCR, suggesting that iron overload contributes to mitochondrial dysfunction. This finding prompted us to examine mitochondrial integrity through both immunoblotting and electron microscopy.

Mitochondrial iron overload is often associated with damage to iron-containing OXPHOS components, of which Complex II component SDHB is particularly sensitive. Indeed, mitochondrial dysfunction in *sgFth1* cells was associated with a marked reduction of SDHB levels and a decrease in phosphorylation of pyruvate dehydrogenase (PDH) complex, phenotypes that can be rescued by overexpression of an *Fth1* cDNA (Fig. 2I). The PDH complex links glycolysis to the TCA cycle through the conversion of pyruvate into acetyl-CoA for use in the mitochondria. It is activated by dephosphorylation the complex under conditions of low ATP, high pyruvate, or high Ca^2+^ (*14*). These data suggest that *sgFth1* cells are responding to cellular stress by modulating PDH but are unable to effectively increase OXPHOS due to damage to electron transport chain proteins. The observation that both of these phenotypes can be rescued by overexpression of an *Fth1* cDNA in high iron conditions implicates free iron as the instigator of both the glycolytic rewiring and mitochondrial damage. Furthermore, it has been established that loss of SDHB protein levels leads to disruption of mitochondrial cristae (*15*, *16*). Consistent with these reports, EM images of iron-treated *sgFth1* cells showed similar mitochondrial content to untreated samples (fig. S2S) but a loss of cristae and outer mitochondrial membrane architecture upon extended treatment (fig. S2T and Fig. 2, J and K). In the control setting, iron treatment resulted in larger mitochondria, which may be consistent with increased OCR (fig. S2T). Overall, we demonstrate that ferritin deficient cells in a high iron environment have altered glycolytic metabolism, impaired mitochondrial function, and decreased proliferation. These findings are consistent with the observed cell-intrinsic reduction of tumor growth in the high-iron environment of the liver.

### Iron-induced metabolic changes drive altered ionic Hhmeostasis and activation of an immunostimulatory cytokine in *Fth1* KO cells

**Fig. 3.**
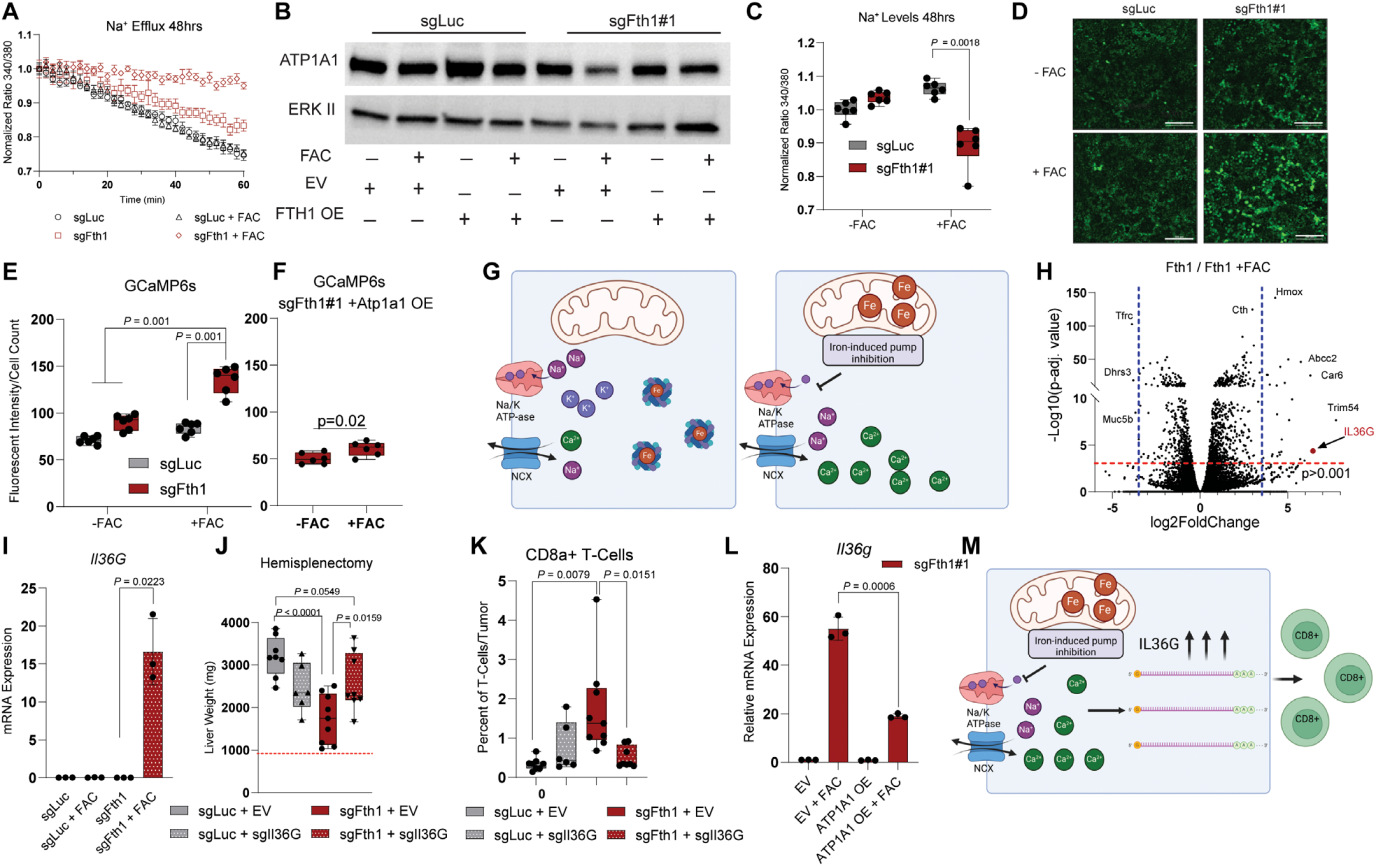
Iron induced ion imbalance mediates anti-tumor immune response via upregulation of IL36G. **A**) Na^+^/K^+^-ATPase activity, *sgLuc* or *sgFth1* cells ±100uM FAC, 48 hours. Na^+^ efflux as surrogate for Na^+^/K^+^-ATPase activity. Data are median ± SEM, six wells, representative experiment. (**B**) Immunoblot, indicated proteins, *sgLuc* and *sgFth1* cells expressing empty vector (EV) or *Fth1* cDNA (FTH1 OE), ± 100uM FAC, 48 hours. (**C**) Total Na^+^, *sgLuc* or *sgFth1* cells ±100uM FAC, 24 hours, Na^+^-specific dye SBFI, 1 hour. in K^+^ free buffer. Data are median ± range, 6 wells, representative experiment. (**D**) Representative images, *sgLuc* or *sgFth1* cells expressing GCaMP6s calcium reporter, ± 100uM FAC, 48 hours. Scale bar, 300uM. (**E**) Quantification of d. Total fluorescent intensity per cell number. Points represent individual wells, *sgLuc* and *sgFth1* cells ±100uM FAC. Data are median ± SD, six wells, representative experiment. (**F**) Total fluorescent intensity as in (E), FTH1 KO, ATP1A1 OE. (**G**) Schematic of iron overload impairing Na^+^/K^+^ ATPase resulting in cytosolic calcium accumulation created in BioRender. Biancur, D. (2026) https://BioRender.com/k52x079. (**H**) Volcano plot, RNAseq, *sgFth1* cells ± FAC. Significance for adjusted *P* value, 0.001 (red line). Significance for Log2 Fold change, 3.5 (blue lines). (**I**) RNAseq, IL36G, *sgLuc* and *sgFth1* ± FAC. Data are mean ± SD, three wells. (**J**) Liver hemisplenectomy metastasis, *sgLuc* or *sgFth1* cells, with EV or sgIL36G (*sgLuc* + EV *n* = 8, *sgLuc* + *sgIl36g n* = 6, *sgFth1* + EV *n* = 9, *sgFth1* + *sgIl36g n* = 8*)*. Data are median ± range, individual tumors. (**K**) Immunohistochemistry quantification, percentage infiltrating CD8a^+^ T-cells, liver metastases from j. Data are median ± range, independent livers/tumors. (**L**) RT-qPCR, IL36G, *sgFth1* cells with EV or *Atp1a1* cDNA (ATP1A1 OE) ± 100uM FAC, 48 hours. Data are mean ± SD, three technical replicates, representative experiment. (**M**) Schematic, iron overload inhibits Na^+^/K^+^-ATPase resulting in *Il36g* transcription created in BioRender. Biancur, D. (2026) https://BioRender.com/tk7p3w4. Statistics, GraphPad Prism. Unpaired, heteroscedastic Student’s t-tests.

The ability of a cell to maintain metabolic homeostasis is intimately linked to the maintenance of ionic balance. Given the metabolic changes described above, we considered whether major ion fluxes are perturbed by loss of the ability to retain iron in ferritin. As we observed lactate accumulation in iron overloaded FTH1 KO cells, we first monitored intracellular pH using a pH responsive dye. In long-term FAC-treated conditions, we observed no acidification in the intracellular pH of either *sgLuc* or *sgFth1* cells (fig. S3A). Surprisingly, *sgFth1* cells treated with a FAC time-course trended toward an alkalinization of the intracellular space, suggesting that these cells may have activated a homeostatic mechanism to maintain cytosolic pH (fig. S3B). Indeed, upon iron overload *sgFth1* cells induce a number of transporters involved in pH homeostasis (fig. S3C). Noting that many of these transporters utilize Na^+^ as a co-transport or counter exchange ion, we investigated Na^+^ handling in our cells. The primary mechanism of Na^+^ efflux is via the Na^+^/K^+^-ATPase (*17*). We confirmed this mechanism in our system by treating cells with Na^+^/K^+^-ATPase inhibitor digitoxin to demonstrate that blocking the Na^+^/K^+^-ATPase reduces Na^+^ efflux (fig. S3, D and E). We then established that Na^+^ efflux was K^+^ dependent by performing a titration of K^+^ and assessing Na^+^ efflux using digitoxin as our positive control for reduced Na^+^ efflux (fig. S3, F and G). Finally, we used Na^+^ ionophores monensin and gramicidin to demonstrate that we could detect Na^+^ increases (fig. S3, H and I) (*18*). Indeed, after 24 hours of FAC treatment both *sgLuc* and *sgFth1* cells had higher intracellular levels of Na^+^ when measured in K^+^ free buffer to block Na^+^/K^+^-ATPase activity (fig. S3J). Remarkably, when K^+^ was reintroduced into the cells, both control and FTH1 KO cells responded by hyperactivating the Na^+^/K^+^-ATPase in FAC-treated conditions, thus leading to a progressive decline in intracellular Na^+^ levels (Fig. 3K). The Na^+^/K^+^-ATPase is one of the major consumers of cellular ATP and accounts for 20–30% of ATP usage under normal cellular conditions (*19*). Based on our observed hyperactivation of the Na^+^/K^+^-ATPase, we speculate that this percentage may be even higher in iron overloaded cells, making the balancing of ions under iron overload a very energetically expensive process. Interestingly, FTH1 KO cells treated with FAC for 48 hours exhibited markedly reduced Na^+^/K^+^-ATPase activity whereas FAC-treated *sgLuc* cells revealed Na^+^ efflux comparable to that of untreated controls (Fig. 3A). We hypothesize that this suppression of the Na^+^/K^+^-ATPase activity in *sgFth1* cells occurs downstream of energy stress caused by perturbed glycolytic and mitochondrial metabolism, oxidation of the ATP1A1 subunit itself, or a combination of these events (*20*, *21*). In line with this idea, *sgFth1* cells under iron overload conditions showed decreased total levels of ATP1A1, the catalytic subunit of the Na^+^/K^+^-ATPase, which was rescued by the addition of *Fth1* cDNA (Fig. 3B). Given the loss of Na^+^/K^+^-ATPase activity in chronically iron overloaded FTH1 KO cells, we were surprised to find that total cellular levels of Na^+^ are decreased upon starvation of K^+^ when compared to controls (Fig. 3C). These data imply an alternative, K^+^ independent, mechanism of Na^+^ efflux. One such mechanism that can be employed under hyperosmotic or Na^+^/K^+^-ATPase inhibited conditions is a process known as NCX reversal (*22*, *23*). The NCX protein family (SLC8A1, SLC8A2, SLC8A3) is a Na^+^/Ca^2+^ exchanger best known for its role in cardiac actional potential. Under normal conditions, NCX operates by allowing Na^+^ influx into the cell in exchange for intracellular Ca^2+^ to balance intracellular Ca^2+^ levels and control cardiac contractility (*24*). When intracellular Na^+^ accumulates, NCX can reverse direction at positive membrane potential, exporting Na^+^ and causing intracellular Ca^2+^ accumulation (*24*). In line with our hypothesis that FTH1 KO cells were likely utilizing NCX as a means of exporting Na^+^, we observed a marked increase in the total cytosolic cellular calcium content when cells were treated with FAC for 48 hours as measured by the cytosolic calcium reporter GCaMP6s (Fig. 3, D and E). We also observed a trend toward *Slc8a2* transcriptional upregulation and increased SLC8A2 protein in iron overloaded FTH1 KO cells (fig. S3, L and M). While these transporters are consistent with adaptation brought on by Na^+^/K^+^-ATPase inhibition, they are likely not the only mechanism by which calcium is accumulating within the cell. We cannot rule out increases of cytosolic calcium driven by calcium stores (endoplasmic reticulum, lysosome, and mitochondria) or decreased calcium extrusion from the cell itself brought on by described metabolic dysfunction. Given that calcium accumulation appears to be downstream of Na^+^/K^+^-ATPase activity and decreased ATP1A1 expression, we exogenously expressed ATP1A1 in FTH1 KO cells to restore ion balance (fig. S3N). Indeed, ATP1A1 overexpression partially rescued GCaMP6s fluorescence in iron overloaded FTH1 KO cells, implying improved Na^+^/K^+^ homeostasis and normalization of calcium flux (Fig. 3F). In summary, these data demonstrate that iron overload in FTH1 KO cells induces alterations in ionic balance, including inhibition of the Na^+^/K^+^-ATPase and cytosolic calcium accumulation (Fig. 3G).

The increase in intracellular calcium is especially intriguing due to its known role in signal transduction, including regulation of inflammatory cytokine production (*25*). We reasoned that elevated intracellular calcium instigated by the high iron environment of the liver may lead to the anti-tumor immune phenotype we identified in Fig. 1. To discover potential immuno-stimulatory changes caused by calcium increase we probed our RNA-seq data from *sgFth1* cells treated with FAC. We discovered that one of the most upregulated transcripts in FTH1 KO cells treated with FAC is the cytokine *Il36g* (also known as *Il1f9* in mice) (Fig. 3H). Interestingly, this transcript was only induced in FTH1 KO conditions when treated with FAC (Fig. 3I). While IL36G is understudied in the context of tumor immunity, a recent publication highlighted the anti-tumor immune properties of overexpressing exogenous IL36G in melanoma (*26*). Iron treatment only stimulated *Il36g* transcript in the FTH1 KO setting (Fig. 3I), so we hypothesized that co-deleting IL36G in FTH1 deficient cells would restore tumor growth in immune-competent animals. Therefore, we generated control and *sgFth1* cell lines additionally infected with an empty vector control or sgRNAs targeting *Il36g*, and these cells were introduced into the liver via the hemisplenectomy metastasis model (fig. S3O). Deletion of IL36G restored the tumorigenic potential of FTH1 deficient cells, indicating that IL36G is required for the anti-tumor immune response of FTH1 deficient cells in the liver (Fig. 3J). Furthermore, when we assayed liver metastases for CD8a^+^ T cell infiltration, we discovered that deletion of IL36G resulted in a marked reduction in tumor infiltrating T-cells (Fig. 3K and fig. S3P). It is currently unclear if CD8a^+^ are participating in the anti-tumor immune response or as a readout of immune cell recruitment to the tumor. Indeed, Il36g was shown to modulate the response of a number of different immune cell populations in vivo and careful evaluation of many immune subtypes will be necessary to understand the response instigated by Il36g (*26*). Considering ATP1A1 overexpression could partially normalize calcium flux, we asked whether upregulation of *Il36g* transcript is mediated by ion imbalance downstream of iron overload, leading to cytosolic calcium accumulation. Indeed, overexpression of ATP1A1 is sufficient to limit *Il36g* induction (Fig. 3L). Taken together, these data demonstrate that iron-induced loss of Na^+^/K^+^-ATPase activity drives Il36g upregulation and incites an anti-tumor immune response in pancreatic cancer liver metastases (Fig. 3M).

### Iron overload drives IL36G expression through inhibition of LKB1 signaling

**Fig. 4.**
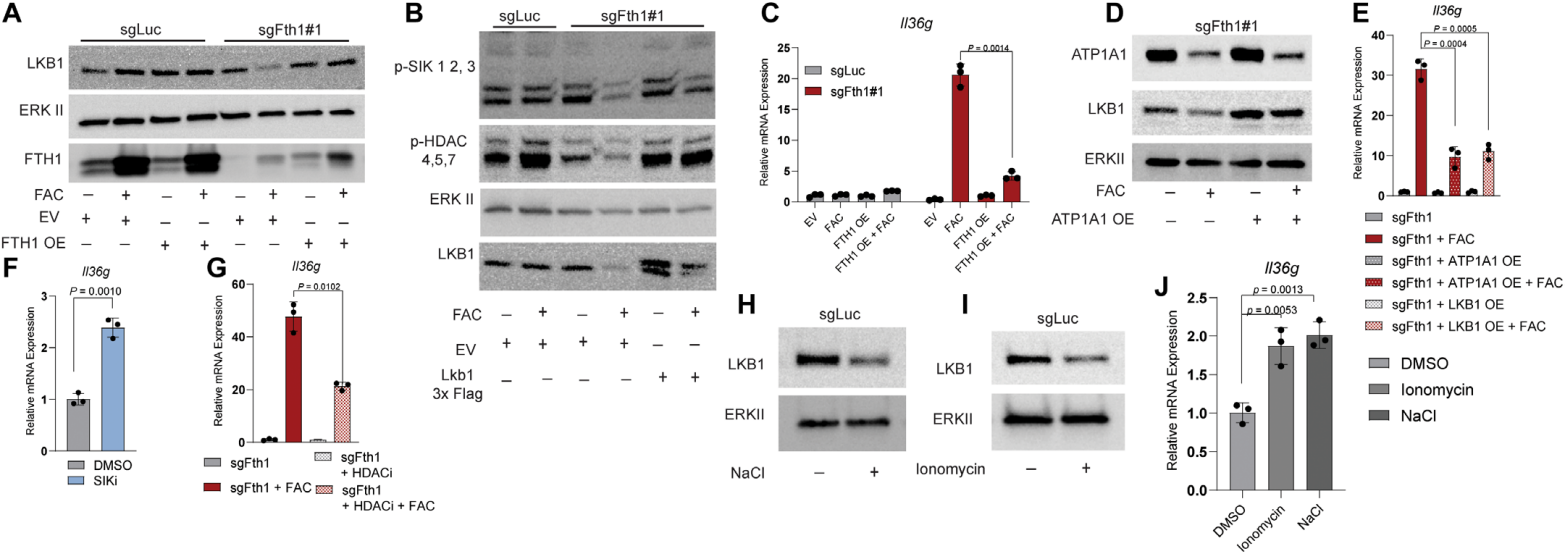
Iron overload drives IL36G expression through inhibition of both SIK signaling. (**A**) Representative immunoblot of *sgLuc* and *sgFth1* cells infected with empty vector (EV) or *Fth1* cDNA (FTH1 OE), ± FAC, 48 hours, probed for LKB1, ERKII, and FTH1. (**B**) Representative immunoblot, *sgLuc* and *sgFth1* cells with EV or LKB1 cDNA (LKB1 3xflag) ± FAC, 48 hours, probed for LKB1, ERKII, phospho-HDAC4,5,7, and phospho-SIK1,2,3. (**C**) RT-qPCR for IL36G in *sgLuc* and *sgFth1* cells with EV or *Fth1* cDNA (FTH1 OE) ± 100uM FAC, 48 hours. Data are mean ± SD, three technical replicates, representative experiment. (**D**) Representative immunoblot, *sgLuc* and *sgFth1* cells with EV or *Atp1a1* cDNA (ATP1A1 OE) ± FAC, 48 hours, probed for ATP1A1, LKB1, and ERKII. (**E**) RT-qPCR for IL36G, *sgLuc* and *sgFth1* cells with EV, *Atp1a1* cDNA (ATP1A1 OE), or *Lkb1* cDNA (LKB1 OE) ± 100uM FAC, 48 hours. Data are mean ± SD, three technical replicates, representative experiment. (**F**) RT-qPCR for IL36G in *sgLuc* and *sgFth1* cells ±100uM FAC, 48 hours with vehicle (DMSO) or HDACi (2uM). Data are mean ± SD, three technical replicates, representative experiment. (**G**) RT-qPCR for IL36G in *sgLuc* or *sgFth1* cells ± vehicle (DMSO) or SIKi (500 nM), 24 hours. Data are mean ± SD, three technical replicates, representative experiment. (**H**) Representative immunoblot of *sgLuc* cells treated with vehicle (DMSO) or ionomycin (1uM), 24 hours and probed for LKB1 and ERK II. (**I**) Representative immunoblot of *sgLuc* cells ± vehicle (DMSO) or NaCl (100 mM), 24 hours, probed for LKB1 and ERKII. (**J**) RT-qPCR for IL36G in *sgLuc* cells ± ionomycin (10uM) or NaCl (100 mM), 24 hours. Data are mean ± SD, three technical replicates, representative experiment.

The loss of FTH1 in a high iron environment promotes anti-tumor effects through perturbation of cellular metabolism and activation anti-tumor immunity. Targeting FTH1 directly is likely not a viable therapeutic strategy to elicit an anti-tumor immune response in pancreatic cancer and would likely only be efficacious in liver metastases; however, we reasoned that insight into the mechanism of *Il36g* upregulation may reveal novel therapeutic opportunities to boost its expression in primary and metastatic PDAC tumors. IL36G is most commonly studied in the context of psoriatic lesions where it promotes inflammatory immune responses (*27*). Indeed, IL36G is a member of the IL-1B family, and we hypothesize that regulation of IL36G may be similar to that of other inflammatory cytokines. Recently, LKB1 loss was found to instigate inflammatory potential through SIK-mediated phosphorylation resulting in nuclear exclusion of class IIa HDACs that modulate chromatin acetylation (*28*, *29*). To test if a similar mechanism could regulate IL36G in PDA, we measured LKB1 protein levels in FTH1 KO cells treated with FAC and observed a reduction in LKB1 protein (Fig. 4A). Consistent with the decrease of LKB1 under iron overload, we observed a marked reduction in both SIK and HDAC phosphorylation that could be rescued by overexpression of 3xFLAG *Lkb1* cDNA (Fig. 4B). Expectedly, LKB1 protein levels protein levels as well as induction of IL36G can be rescued by FTH1 overexpression (Fig. 4, A and C). We then considered whether ion imbalance is driving this reduced LKB1 protein levels. Indeed, overexpression of ATP1A1 rescues the decrease in LKB1 protein levels in FAC treated samples (Fig. 4D). Excitingly, in addition to rescuing the protein levels, both a 3xFLAG *Lkb1* cDNA and Atp1a1 overexpression results in the marked reduction of *Il36g* mRNA levels (Fig. 4E). Taken together, these data demonstrate that ion dysfunction mediated by loss of Na^+^/K^+^ ATPase activity results in the reduction of LKB1 protein levels disrupting a signaling cascade leading to increased Il36g production.

We then considered whether modulating this LKB1 signaling axis access impacts *Il36g* expression independent of FAC or ion imbalance. Interestingly, inhibition of SIK, which should phenocopy LKB1 loss, is sufficient to induce *Il36g* mRNA independent of FAC treatment and FTH1 deficiency (Fig. 4F). Furthermore, treatment with a Class IIa HDAC inhibitor reduces the levels of IL36G induction, implying that Class IIa HDAC activity is required for this phenotype (Fig. 4G).

Given that *Il36g* can be induced through disrupting the signaling cascade downstream of LKB1, we asked whether other ion disrupting treatments could modulate LKB1 protein level. Indeed, treatment with ionomycin (calcium ionophore) or NaCl (hyperosmotic conditions result in increased calcium abundance) are sufficient to decrease LKB1 protein levels (Fig. 4, H and I). Furthermore, both ionomycin and NaCl treatment were sufficient to drive *Il36g* expression in these cells further supporting the notion the ion imbalance prevents translation of Lkb1 leading to loss LKB1 protein and signaling disruption (Fig. 4J).

**Fig. 5.**
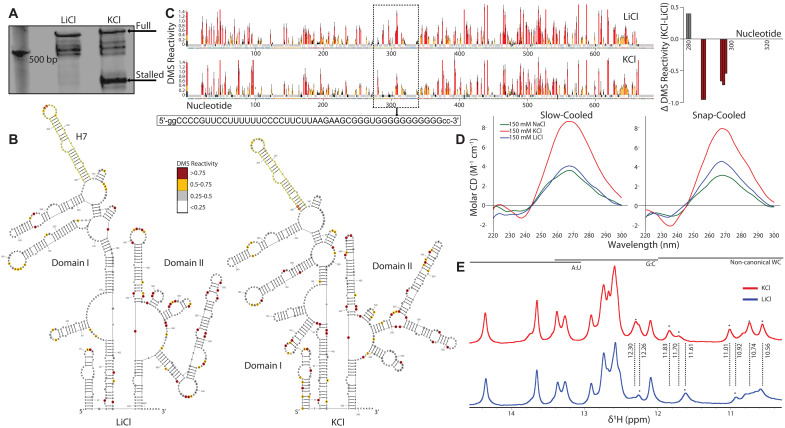
Identification of RNA G-Quadruplex in LKB1. (**A**) Primers designed to the 3′ end of LKB1 5′UTR were used for foot-printing the mRNA in either LiCl (control) or KCl (secondary structure inducing) conditions. The presence of a secondary structure in the LKB1 5′UTR will prevent RT progression. Running the cDNA from RT foot-printing on a denaturing gel highlights a secondary structure present only in KCl conditions that prevents RT progression implying the presence of a secondary structure that can affect LKB1 translation efficiency. (**B**) LKB1 5′UTR secondary structures determined via chemical probing. Predicted G4 (H7) is highlighted in yellow. (**C**) Reactivity profiles of LKB1 5′UTR refolded in the presence of LiCl (top) and KCl (bottom). The region proposed to contain a G-quadruplex is boxed and the sequence is highlighted. The change in reactivity of nucleotides within the boxed region corresponding to the G4 is compared, where -Δ indicates less reactivity when refolded in the presence of KCl and + Δ indicates higher reactivity. Region proposed to contain the quadruplex is boxed. Sequence of the Gg-quadruplex is highlighted. (**D**) CD spectra for slow-cooled (left) and snap-cooled (right) RNA in the presence of 150 mM NaCl, KCl, *or* LiCl. CD profiles show positive peaks at ~265 nm that are characteristic of G4 formation and are most emphasized in the KCl conditions. (**E**) 1D 1H NMR spectra of the imino region of LKB1 in NMR buffer containing 15 mM NaP pH 6.5, 10 mM MgCl_2_, 10% D_2_O, and *either* 150 mM KCl or 150 mM LiCl. Upfield resonances are more pronounced in the KCl condition, suggesting the formation of a structured quadruplex.

Because we did not observe changes to *Lkb1* mRNA levels in the aforementioned conditions where LKB1 protein was lost (fig. S5A), we considered whether the reduction in LKB1 protein levels is caused by increased LKB1 turnover or decreased translational efficiency. To differentiate between these possibilities, we treated FTH1 knockout cells with FAC to decrease LKB1 levels and added either chloroquine (CQ) to block autophagic protein turnover or Bortezomib to block proteasome-mediated turnover. Interestingly, both CQ and Bortezomib did not increase LKB1 levels suggesting that there was not increased protein turnover in FAC overloaded conditions (fig. S5, B to D). Moreover, we noted that exogenous 3xFlag-tagged *Lkb1*, which has no 5′UTR, is not decreased under FAC treatment (Fig. 4B), a condition in which the endogenous protein level is decreased. To consider mechanisms that modulate *Lkb1* translational efficiency, we analyzed the *Lbk1* mRNA for possible regulatory elements. Through this in silico analysis, we discovered that the relatively long 5′UTR of *Lkb1* contains a putative RNA G-quadruplex (fig. S4F). This was noteworthy considering that RNA G-quadruplexes, especially in the 5′UTR, have well-established roles in translational efficiency (*30*, *31*). While K^+^ is the prototypical ion involved in quadruplex formation, it was recently reported that calcium influx could accelerate RNA G-quadruplex formation (*20*, *32*). We reasoned that ionic perturbations downstream of iron overload may create a favorable ionic environment to promote secondary structure formation through interaction with the RNA itself or through regulating proteins that resolve quadruplexes (*32*). To test if the *Lkb1* 5′UTR contained an element capable of creating a secondary structure and preventing translation, we performed a reverse transcriptase (RT) footprinting assay on in vitro transcribed mRNA under both LiCl (control) and KCl (quadruplex inducing) conditions (Fig. 5A). Our data revealed a stalled cDNA product in the 5′UTR of *Lkb1* under KCl conditions but not under LiCl conditions. In contrast, a control 5′UTR with no predicted quadruplex structures did not show any RT stalling in either LiCl or KCl conditions (fig. S5F). We mapped the quadruplex secondary structure of LKB1 and ascertain G-quadruplex formation using dimethyl sulfate mutational profiling and sequencing (DMS-MaPseq), structural chemical probing, circular dichroism (CD) and nuclear magnetic resonance (NMR) spectroscopy in the presence of either LiCl or KCl (Fig. 5, B to E). DMS-MaPseq experiments reveal that LKB1 is intricately structured under both buffer conditions, and the reactivity is in good agreement with the predicted 2D structures, with single-stranded regions (e.g., apical and internal loops, and bulges) showing increased reactivity relative to base-paired stem motifs (Fig. 5B). Upon comparing the KCl and LiCl reactivity profiles, we noted differences localized to two stretches nt 280–339 and nt 375–400. Consistently, the changes in reactivity do not appear in NaCl treated conditions (fig. S5G). Notably the differential reactivity between nt 280–339 occurs in the same region where we observed stalling in the RT footprinting experiments and overlaps with the RNA G4 predicted in silico (Fig. 5C) These nucleotides are predicted to form an extended hairpin (HP7) rich in guanosines. We isolated HP7 and carried out CD and 1D ^1^H NMR experiments to further investigate the cation-dependence of the LKB1 HP7.

The CD signal of the LKB1 HP7 in KCl was increased relative to the NaCl and LiCl buffer conditions (Fig. 5D). Notably, this trend was observed regardless of the refolding protocol (fig. S5H) (see also Methods; thawed, slow-cooled, or snap-cooled). The strong difference in peak heights suggests that K^+^ ions strongly coordinate the G-quadruplexes, while the smaller size of Li^+^ leads to a loss of coordination and collapse of the G-quadruplex structure - incidentally a loss of measured absorbance in the CD experiments. 1D ^1^H NMR spectra of LKB1 under the KCl and LiCl conditions reveals the upfield imino resonances (~10–12 ppm; a region where non-canonical base pair signals resonate) to be more pronounced in the KCl condition, with prominent and narrow chemical shifts seen at 10.56 ppm, 10.74 ppm, and 11.01 ppm (Fig. 5E). Other peaks are also present with higher relative intensity compared to the LiCl condition, such as that at 11.83 ppm and 12.30 ppm. These data strongly suggest that K^+^ is responsible for enabling coordination of the G-quadruplex into an ordered structure.

Taken together, these data demonstrate that the 5′UTR of *Lkb1* contains an RNA G-quadruplex that is sensitive to the ionic environment and support the hypothesis that LKB1 protein levels are suppressed upon iron overload through this mechanism. We propose that modulation of this mRNA regulatory element is a mechanism by which cells can control LKB1 mediated signaling cascades when under ionic stress. Considering LKB1 is such a critical component to metabolic homeostasis, more work into this area will shed light on the contexts in which cells can regulate metabolism under stress via this mechanism.

### Iron overload drives *Il36g* expression through disruption of NMD

**Fig. 6.**
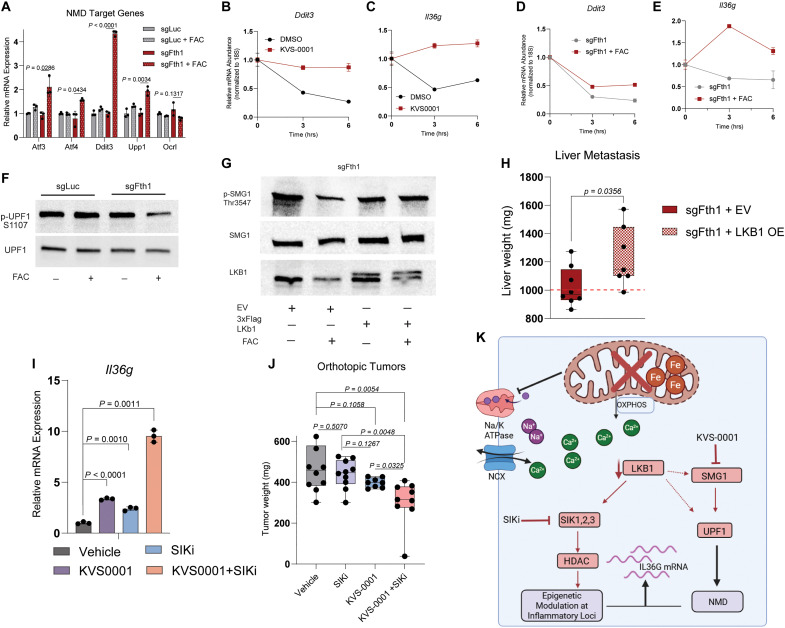
Iron overload stabilizes IL36G mRNA through inhibition of NMD signaling. (**A**) RNA sequencing, *sgLuc* and *sgFth1* cells ± FAC, NMD target genes Atf3, Atf4, Ddit3, and Upp1. Ocrl is a non-NMD control. Data are mean ± SD, individual wells. (**B** to **E**) RNA stability assay, *sgLuc* or *sgFth1* cells or those expressing CRISPRa IL36G ± vehicle (DMSO), KVS0001 (0.5uM), 24 hours, or FAC (100uM, 48 hours). 3 and 6 hours prior to collection, actinomycin D added. Data are mean ± SD, three [(B) and (C)] or two [(D) and (E)] technical replicates, representative experiment. (**F**) Representative immunoblot, *sgLuc* and *sgFth1* cells ± FAC, 48 hours, probed for phospho-UPF1 and UPF1. (**G**) Representative immunoblot, sgFth1 cells with EV or 3xflag-tagged Lkb1, probed for p-SMG1 Thr3547, SMG1, and LKB1. (**H**) Liver hemisplenectomy metastasis model, *sgFth1* cells infected with EV or 3xflag-tagged Lkb1, *sgFth1* + EV *n* = 8, *sgFth1* + *Lkb1 n* = 7. Data are median ± range of individual tumors. (**I**) RT-qPCR for *Il36, sgLuc* cells treated with either SIKi or KVS-0001. Data are mean ± SD, three technical replicates, representative experiment. (**J**) Pancreatic orthotopic model, sgLuc cells, NMDi and SIKi alone or in combination. (Vehicle, *n* = 9; SIKi, *n* = 10; KVS-0001, *n* = 8; SIKi + KVS-0001, *n* = 9). (**K**) Schematic for how iron-induced mitochondrial dysfunction drives calcium accumulation to decrease the levels of LKB1 protein (red arrow) created in BioRender. Biancur, D. (2026) https://BioRender.com/jr5smmk. Decreased LKB1 leads to reduced phosphorylation of SIKs and enhanced HDAC activity to promote IL36G transcription. Lkb1 may also signal through the NMD pathway via SMG1 and/or UPF1 (dotted line). Lkb1 loss reduces NDM activity, stabilizing Il36 and other transcripts.

While the data described above begin to explain the induction of inflammatory cytokines like IL36G under ionic stress, we noted that *Il36g* induction upon SIK inhibition is modest when compared to iron overload. We therefore asked whether there might exist additional mechanisms by which iron overload impacts *Il36g* transcript abundance. We noticed that a number of nonsense-mediated mRNA decay (NMD) target genes were increased only upon iron overload, conditions in which *Il36g* is strongly upregulated (Fig. 6A) (*33*). NMD is a cellular homeostatic mechanism that degrades roughly 5–10% of endogenous mRNA in addition to mRNAs harboring a premature termination codon (*33*, *34*). While *Il36g* mRNA is not an established NMD target, these data suggested that *Il36g* mRNA stability may be regulated by the NMD pathway. To test this, we performed an RNA stability time course in *sgFth1* cells treated with FAC and an NMD inhibitor. We used CRISPRa upregulation of *Il36g* to uncouple transcription from RNA stability and observed remarkable stabilization of both canonical NMD transcript *Ddit3* and *Il36g* upon treatment with KVS-0001, an established NMD inhibitor (Fig. 6, B and C). Moreover, in FTH1 KO cells *Ddit3* and *Il36g* are both stabilized by FAC treatment, demonstrating that iron overload inhibits NMD (Fig. 6, D and E). The canonical NMD pathway is regulated in part by the phosphorylation of the helicase UPF1 by the kinase SMG1, which promotes interactions with other NMD components thereby triggering NMD (*33*, *35*). Accordingly, FTH1 KO cells treated with FAC showed a marked reduction in the phosphorylation of core NMD component UPF1 (Fig. 6F). NMD inhibition via KVS-0001 treatment resulted in a reduction in p-SMG1 to a similar degree (fig. S6A). Taken together, these data demonstrate that NMD is inhibited under iron overload, possibly through disruption of SMG1 phosphorylation, and that *Il36g* is stabilized upon NMD inhibition.

We then considered whether the observed NMD inhibition was functionally linked to reduction in LKB1 protein levels. High-throughput phospho-proteomic data suggests that an LKB1 phosphorylation motif exists on SMG1 (fig. S6B) (*36*, *37*). In line with this observation, overexpressing 3xFlag-tagged *Lkb1* cDNA in iron overloaded *sgFth1* cells restored SMG1 phosphorylation (Fig. 6G). Importantly, LKB1 overexpression significantly restored growth of FTH1 knockout cells in the hemisplenectomy model of liver metastasis. These data suggest that iron overload instigated by the liver microenvironment reduces LKB1 protein expression in vivo and reconstituting *Lkb1* without the 5′UTR restores Lkb1 protein levels thereby suppressing the anti-tumor immune response mediated by Lkb1 loss.

Based on the above analyses, we propose that LKB1 impacts *Il36g* expression and therefore tumorigenesis through two distinct downstream regulatory axes: SIK and Class IIa HDAC-mediated modulation of *Il36g* transcription and NMD-mediated modulation of *Il36g* mRNA stability. We therefore asked whether targeting both regulatory axes could cooperate to promote *Il36g* induction. Indeed, treatment with both KVS-0001 and SIKi synergistically increases *Il36g* mRNA, an effect similar in magnitude to iron overload in *sgFth1* cells. While the high iron environment of the liver was required to induce *Il36g* in FTH1 KO cells in the hemisplenectomy model, we were curious as to whether or not we could phenocopy this observation in the pancreas with inhibitors. We therefore functionally tested these findings in an orthotopic model of pancreatic cancer. While both inhibitors fail to produce a significant anti-tumor response alone, the combination was strongly additive and promoted a robust anti-tumor response in a primary pancreatic cancer model (Fig. 6J). We were then interested in whether or not these findings would be applicable to human PDA cell lines and human disease. To this end, we demonstrate that in PaTu-8902 with FTH1 KO there is a significant growth disadvantage when treated with high iron (fig. S6, C and D). Furthermore, high iron induces expression of *IL36G* in FTH1 KO cells (fig. S6E). The induction of *IL36G* can be phenocopied by adding NMD inhibitor KVS-0001 suggesting that *IL36G* is an NMD responsive transcript in human PDA cells (fig. S6F). Importantly, we also observe in iron overloaded FTH1 KO cells that LKB1 protein is decreased as expected thus suggesting that the identified signaling cascades into SIK and NMD are likely perturbed in human cells as well (fig. S6G). Excitingly, alterations in critical NMD kinase SMG1 is predictive of survival in pancreatic cancer patients further suggesting that the pathway is active in human disease and a novel therapeutic opportunity (fig. S6F). Finally, we also map an RNA G-quadruplex to the 5′UTR of human LKB1 as demonstrated by chemical probing experiments (fig. S7, A to F). Differential reactivity was observed in the human LKB1 KCl and LiCl reactivity profiles, with the KCl profile showing lower overall reactivity consistent with KCl promoting stability (fig. S7B). Human LKB1 is similarly intricately structured, and changes in reactivity were observed for several regions corresponding to computationally predicted G-quadruplexes (fig. S7C). These results support that G-quadruplex formation is a conserved mechanism of regulation between human and mouse LKB1.

Based on our findings, we present our working model whereby iron-induced ion dyshomeostasis results in LKB1 translational repression mediated by a G-quadruplex in the 5′UTR, resulting in perturbation in both the SIK signaling pathway and the NMD signaling pathway. Together, these pathways converge on *Il36g* transcription and stabilization and subsequent stimulation of anti-tumor immune responses (Fig. 6K). Additional work in this arena such as the development of more potent and selective SIK and NMD inhibitors may provide a novel and exciting way of activating anti-tumor immunity in pancreatic cancer.

## DISCUSSION

While it is well-established that pancreatic cancer cells depend on altered metabolic networks, the full extent of the role of tumor metabolism to drive growth and metastasis is an area of ongoing investigation (*38*, *39*). Through in vivo genetic screening with a metabolism-focused sgRNA library, we have identified genes critical for promoting the growth of PDA tumors in the metastatic liver microenvironment. Our data highlight FTH1 as the most striking example of a liver-specific metabolic liability. To this end, loss of FTH1 results in a growth disadvantage specifically in the context of the liver, potentially due to the high iron content found in this tissue. We demonstrate that iron overload in the context of FTH1 KO leads to oxidative stress, perturbations in upper glycolysis, and mitochondrial dysfunction. Excess free iron and disruption of these two critical metabolic pathways ultimately converge on the inability to maintain ion homeostasis.

LKB1 is a master kinase that modulates cellular metabolism through phosphorylation of AMPK-related kinases under energetic stress to inhibit anabolic pathways (*40*). While LKB1 is more commonly studied as a tumor suppressor in the context of lung cancer, we find that reduction of LKB1 under ionic stress results in the transcriptional upregulation of immune-stimulatory cytokine IL36G via a recently delineated SIK-HDAC signaling axis and inhibition of NMD (*28*). Mechanistically, we demonstrate the presence of an RNA G-quadruplex in the 5′UTR of LKB1 that controls LKB1 protein levels upon iron overload, consistent with this regulation. These data point to a novel mechanism of metabolic control whereby cells under ionic stress can suppress LKB1 protein levels. Indeed, disruption of the intracellular ionic environment may influence the translation of multiple mRNAs containing RNA G-quadruplexes.

There are multiple mechanisms by which altered ionic homeostasis could control RNA G-quadruplexes. For example, intracellular calcium may regulate RNA helicases that are responsible for unwinding RNA G-quadruplexes, thus promoting translation. Indeed, the key NMD regulator SMG1 is known to interact with RNA helicases, and it is tempting to speculate that a feed-forward loop exists whereby an RNA helicase unwinds the *Lkb1* G-quadruplex, reducing LKB1-dependent phosphorylation of SMG1 and further inhibiting NMD.

Given the tumor suppressive function of LKB1, it also stands to reason that LOF mutations in RNA helicases would prevent RNA G-quadruplexes unwinding. Such mutations might provide an alternative mechanism to suppress LKB1 and promote tumorigenesis in other contexts, potentially influencing treatment paradigms. Furthermore, mutations in G-quadruplexes within UTRs may impact regulation of LKB1 or other genes. Future studies into LKB1 translational regulation may reveal other interacting partners in the helicase and NMD family of proteins as well as identify other inflammatory cytokines or key transcriptional programs regulated by acute loss of LKB1 or disruption of ion homeostasis.

Our findings suggest a promising new treatment strategy for controlling the PDA cytokine milieu by modulating intracellular ionic fluxes. Indeed, along with FTH1, one of the top hits in our screen for liver metastasis-specific regulators is Na^+^/K^+^-ATPase subunit ATP1A1. The Na^+^/K^+^-ATPase inhibitor digoxin was recently shown to have anti-tumor properties and reduce circulating tumor cells in breast cancer patients (*41*). We speculate that loss of ATP1A1 in a high iron environment may trigger calcium accumulation and decrease LKB1 translation. We contend that, in addition to decreasing metastasis, treating certain cancer types, such as pancreatic cancer, with digoxin may invoke an immune response through inhibition of the LKB1-SIK and LKB1-SMG1 axes.

While our findings indicate that disruption of the LKB1 signaling axis creates an anti-tumor immune environment in the context of pancreatic cancer, loss of LKB1 signaling may also have pro-inflammatory roles in other diseases such as psoriasis. Indeed, IL36G is implicated in promoting psoriatic lesions (*27*). Beyond LKB1, future investigation into the roles of ion homeostasis controlling protein abundance of other critical signaling regulators may reveal additional important cellular homeostatic mechanisms.

We discovered that disrupted ion homeostasis inhibits NMD with evidence that this may be regulated by LKB1. While NMD has a fundamental role in cellular homeostasis, its role in cancer remains poorly defined (*35*). Tumors exhibit upregulation of NMD machinery to degrade transcripts harboring nonsense mutations, which are highly prevalent in cancer (*35*). Indeed, NMD prevents the production of truncated proteins that may otherwise interfere with normal protein function, cause ER stress, or lead to the production of neoepitopes and immune activation. Thus, tumor cells can hijack the NMD machinery to promote unrestricted growth and immune evasion. Alternatively, certain types of cancer have somatic mutations within *UPF1* or downregulated UPF1 expression through promoter methylation. For example, inflammatory myofibroblastic tumors present with reduced NMD activity resulting in hyperactive NF-KB signaling and a characteristic inflammatory microenvironment (*42*). Taken together, these data suggest that the role of NMD in cancer is nuanced and context-dependent. Here we demonstrate that inhibition of NMD activates an anti-tumor immune response through the stabilization of *Il36g* mRNA. We speculate that the accumulation of neoepitopes caused by readthrough of premature termination codons may synergize with the stabilization of anti-tumor cytokines to promote potent anti-tumor immunity upon NMD inhibition.

While directly targeting FTH1 may not be feasible in PDA, we discovered two targetable signaling nodes disrupted by ion dyshomeostasis downstream of FTH1 loss whose combined inhibition can induce potent *Il36g* induction. Thus, there may be therapeutic potential in targeting SIK in combination with an NMD inhibitor in human PDA. Our data offer strong rationale for exploring the combined therapeutic potential of disrupting these pathways in PDA and potentially other cancers.

## MATERIALS AND METHODS

### Cell lines

Primary mouse PDAC line (HY19636 and HY15549) was generated from tumors from B6 genetically engineered mice (LSL-KrasG12D; p53 L/+, Ptf1a-Cre+) as previously described (*43*). Cell lines were authenticated by periodic fingerprinting as well as visual inspection and carefully maintained in a centralized cell bank. All cell lines were tested routinely, and before all screening efforts, for mycoplasma contamination by PCR. Cell lines were cultured in DMEM (Corning 10–017-CV) with 10% fetal bovine serum (FBS) (Atlanta Biologicals S11550H), and 1% Penicillin/Streptomycin (Thermo, 15140122). These cells were genetically modified for loss-of-function studies using CRISPR/Cas9. Cell lines were also modified with cDNA constructs using lentiviral expression systems.

### Stable cell lines

Stable knockout of FTH1 in murine cells was achieved using 2 independent sgRNAs. Control cells were generated using guides targeting Luciferase. Stable knockout of IL36G in murine cells was achieved using sgRNA from a previously published dataset (*44*). Control cells were generated by using pLentiCRISPRv2 empty vector virus for double infection. All sgRNAs were cloned into pLentiCRISPRv2 (Addgene, plasmid #52961, 83480) and sequence verified prior to viral transduction.

*sgLuc* F 5′-CACCGACAACTTTACCGACCGCGCC-3′

*sgLuc* R 5′-AAACGGCGCGGTCGGTAAAGTTGTC-3′

*sgFth1*#1 F 5′-CACCGACGTCTATCTGTCTATGGTG-3′

*sgFth1*#1 R 5′-AAACGGAGAGCGGGCTGAATGCAAC-3′

*sgFth1*#2 F 5′-CACCGTTGCATTCAGCCCGCTCTCC-3′

*sgFth1*#2 R 5′-AAACCACCATAGACAGATAGACGTC-3′

*sgIl36g* F 5′-CACCGCGAAGCCACAGAGTAACCCC-3′

*sgIl36g* R 5′-AAACGGGGTTACTCTGTGGCTTCGC-3′

Human

sgFth1#1 F 5′-caccgCACCATGGACAGGTAAACGT-3′

sgFth1#1 R 5′-aaacACGTTTACCTGTCCATGGTG-3′

The generation of lentivirus was performed by transfecting 293 T cells with lentiviral vector (plentiCRISPRv2) and packaging plasmids psPAX2 (Addgene, plasmid #12260) and pMD2.G (Addgene, plasmid #12259) at a 0.5:0.25:0.25 ratio with polyethylenimine (PEI). Viral supernatant was collected 48 hours. after transfection. Filtered viral supernatant and polybrene (8 μg/ml) was used to infect target cells. Cells were then subject to antibiotic selection until control plates (uninfected cells) were dead. Cells were selected with 2 μg/ml puromycin for 2 days, 10 μg/ml blasticidin for 10 days, or 300 μg/ml hygromycin for 10 days.

pLVX-IRES-Hygro_GCaMP6s (Addgene plasmid #164591) and pLVX-puro_GCaMP6s (Addgene plasmid #164589) were generous gifts from Joseph Hyser. Lentivirus was generated as described above and target cells infected with reporter constructs. The cell lines were then sorted using fluorescence assisted flow cytometry (FACS) for median expression. *Atp1a1* cDNA was generated by TWIST Biosciences and cloned into an expression construct using the Gateway cloning system as per manufacturer instructions. The *Atp1a1* cDNA was expressed in pLenti CMV Blast DEST (706–1) (Addgene plasmid #17451), a generation gift from Eric Campeau, Paul Kaufman. *Lkb1* cDNA was generated by TWIST Biosciences and cloned into expression construct using the Gateway cloning system as per manufacturer instructions. The *Lkb1* cDNA was expressed in pLenti6.2-ccdB-3xFLAG-V5 (Addgene plasmid #87071), a generation gift from Susan Lindquist, Mikko Taipale.

### Mouse experiments

The liver metastasis surgery was performed as previously described (*45*). Briefly, mice were anaesthetized by intraperitoneal injection of ketamine (120 mg/kg) and xylazine (10 mg/kg). A small incision was made on the upper left quadrant of the abdomen and the spleen exposed. For intrasplenic injection, 1 × 10^6^ cells were suspended in 100 μl HBSS and then drawn into an insulin syringe (28-gauge needle, BD 329461). The externalized spleen was divided by ligating clips (Teleflex, 002200), and cells were injected into the hemispleen. After injection, splenic vein was ligated with ligating clips (Teleflex, 001200) at the hilum of the spleen, and then the hemispleen was removed. After the procedures, the peritoneum was closed with a 3–0 VICRYL VIOLET suture (Ethicon, J311H), and the skin was closed using the BD AutoClip Wound Closing System (BD). The liver metastasis screen was performed in 53 C57/Bl6 mice and liver metastases were grown over the course of 14 days where they harvested, pooled, and sequenced. Mice were treated with buprenorphine every 12 hours after surgery for 48 hours. The mice were monitored daily for signs of distress including jaundice, agonal breathing, or poor body posture.

Liver metastasis validation experiments in both C57BL/6 and NSG mice were injected with 2x10^5 cells of either control (*sgLuc*) or knockout (*sgFth1*).

Pancreas orthotopic and flank xenografts were established as follows. Briefly, 7–8-week-old female C57/BL6 (C57/BL6) were orthotopically injected with tumor cells into the pancreas or subcutaneously injected into the flank. For orthotopic xenografts, mice were anaesthetized with ketamine (120 mg/kg) and xylazine (10 mg/kg) before surgery. HY19636 cells (2x10^4) expressing control (*sgLuc*) or *sgFth1* sgRNA were suspended in 20 μl of 50% growth factor-reduced Matrigel (BD Science) and HBSS, and injected into the pancreas. Any error during injection was recorded as a failed injection and the mouse not used in the experiment. After the procedures, the peritoneum was closed with a 3–0 VICRYL VIOLET suture (Ethicon, J311H), and the skin was closed using the BD AutoClip Wound Closing System (BD). Mice were treated with buprenorphine every 12 hours after surgery for 48 hours. For flank tumors, either C57BL/6 or athymic nude mice were injected with HY19636 cells (1x10^5) in 100 μl of growth factor-reduced Matrigel. The maximum tumor size was 2 cm, which was not exceeded during experiments.

Mice for liver direct injection experiments were anaesthetized by intraperitoneal injection of ketamine (120 mg/kg) and xylazine (10 mg/kg). A small incision was made on the upper left quadrant of the mouse. The liver was gently exposed using cotton swabs. HY119636 cells (5x10^4) were suspended in 50 μl of 50% growth-factor reduced Matrigel and HBSS and injected into the liver lobe. The liver was cauterized as the injection site with a Bovie electro-cautery device to prevent excessive bleeding immediately after the syringe was removed from the liver. The liver was gently returned to the abdomen. After the procedures, the peritoneum was closed with a 3–0 VICRYL VIOLET suture (Ethicon, J311H), and the skin was closed using the BD AutoClip Wound Closing System (BD).

Orthotopic tumors were dosed with 30 mg/kg KVS-0001 and 20 mg/kg of YKL-05-099. Both compounds were delivered IP and dissolved 0.5% Dimethyl sulfoxide (DMSO) (Sigma Aldrich, USA, Cat #C6295), 10% cremaphor (Sigma Aldrich, USA, Cat #C5135) and in normal saline.

All animal studies were approved by the NYULMC IACUC (Institutional Animal Care and Use Committee, Protocol #IA16–00507).

### Liver metastasis screen

Mouse metabolism library was generated as previously described (*38*). The library consists of optimized sgRNA’s and controls targeting 2918 metabolic genes. The metabolic gene list was generated by compiling lists from previous human metabolic screens and mapping these genes to the mouse homologs with the addition of some manually curated genes. All sgRNA’s were developed using established algorithms to optimize on-target and minimize off-target genome editing High titer lentivirus was generated from the mouse metabolism library and cells infected at a multiplicity of integration (MOI) of 0.3. HY19636 cells were infected with previously tittered virus with 8 μg/ml of polybrene (Sigma, TR-1003) by spin infection (2 hours, 30°C, 2000 RPM). After cells have settled on plates (1–2 hours), media was changed and cells allowed to grow overnight. The following day cells were trypsinized and plated in puromycin containing media and selected for 2 days (a time point where uninfected control cells were dead). Immediately after selection cells were split for the following experiments: cells were collected to assess library representation at time = 0 or used in the hemisplenectomy liver metastasis model in C57/BL6 mice.

### CRISPR sgRNA sequencing

Cell pellets from mouse tumors was created by pooling tumors detailed below. Animals were sacrificed and whole livers were harvested and weighed prior to mechanical digestion in 50 ml conical tubes with one liver per tube. DMEM with 2% FBS was then added to the livers so that liver/tumors will be resuspended at a concentration of 0.1 mg/ml. Liver tumor mixtures were then carefully and thoroughly homogenized with scissors on ice trying to get as close to a single cell suspension as possible. Once liver/tumor mixtures were homogenized, 1.6 ml of homogenate from each mouse was added to a 15 ml conical tube, pooling all the tumors from a single cage. This was then spun down, supernatant aspirated, and tumor cell pellet frozen until DNA extraction. DNA from cell/tumor pellets was extracted using the QIAGEN Blood Maxi Kit as per manufacturer’s instructions. PCR was used to amplify the sgRNA region followed by sequencing on an Illumina HiSeq to determine sgRNA abundance.

### Data normalization

Reads per million (RPM) values were calculated for each sample by dividing sgRNA count values for a given sample by the total counts for that sample. These values were then multiplied by 10^6^, to obtain the RPM value for the given sgRNA. The log2(RPM + 1) value was then calculated for each sgRNA. Following this procedure, replicates were then collapsed down by taking the median value. The log fold change value (LFC) was then calculated by subtracting the corresponding early time-point of infection, referred to as the Full Library Representation. For each sample, the median of the individual sgRNAs from the negative control genes was then subtracted from each of the LFC values. Finally, each LFC value was divided by the magnitude of the median of the LFC values of the individual sgRNAs for the positive control genes.

### Data quality control

Distributions of the log2(RPM + 1) values were generated to look at relative drop out of total guide counts relative to condition and empirical cumulative distribution functions, ECDF, were used to ensure that all guides were present in the initial library. ECDFs were created using the ECDF function in the base R stats package (R version 3.6.1).

### STARS analysis

The STARS algorithm version 1.3 was used to provide *P* value, FDR, and q-value estimations for the gene LFC scores (*46*). The Normalized LFC data at the sgRNA level, as described in Data Normalization, was provided. The null distribution was created with a threshold of 60, 1000 iterations, and excluding the first ranked perturbation. STARS was then subsequently run with a threshold of 0.6 in both the positive and negative directions on the normalized log fold change data. The positive and negative runs for a given condition were combined by assigning STARS scores from the negative direction a negative sign and then merging the lists. Conflicts in this merge were resolved by either: taking the corresponding STARS score and statistics associated with the higher *P* value if both were insignificant or taking STARS score and statistics associated with the statistically significant result of a *P* value less than 0.05 and an FDR of less than 0.25.

### Cell proliferation assays

For 2D growth assays, HY19636 and HY15549 cells were plated in 24-well plates at 2,500 cells per well in 0.5 ml of media. For iron overload models, ferric ammonium citrate (FAC,) was added to the wells at the indicated concentrations in the experiment. For most studies, 100 μM of FAC was used to induced iron overload unless otherwise stated. *sgLuc* or *sgFth1* cells were plated into media containing FAC and proliferation assessed over 4–5 days. For Trolox rescue experiments, 2500 cells per well were seeded into media containing vehicle (DMSO) or Trolox (10 μM) either with or without FAC treatment. To assess growth, cells were fixed in the plates using 10% formalin for 15 minutes. The formalin was then aspirated and cells stained with 0.1% crystal violet solution for 10 minutes. The crystal violet was removed and the plates gently washed with water and allowed to dry. Quantification of crystal violet was performed as previously descried. Briefly, 10% acetic acid solution was added to the wells to extract the dye and absorbance read at 595 on the Sprectramax M3 (molecular devices).

For 3D growth assays, HY19636 cells were plated in ultra-low attachment 96-well plates at 2,500 cells per well in 100 μl of media containing 5% growth-factor reduced Matrigel. The cells were then grown over the course of 3 days and growth assessed using cell-titer glo (CTG) reagents. Briefly, 100 μl of CTG was added to the wells of the plate and gently pipetted to break up spheroids. The cells were incubated in CTG solution for 10 min before being transferred to a white-wall 96 well plate. The luminescence was measured using the Spectramax M3 (Molecular Devices).

### Na^+^ flux assays

Cells were cultured in 96-well white-walled plates at 37°C until they reached 80–90% confluence. Cells were either pre-treated with 100uM FAC for 24 or 48 hours prior to assay. Cells were loaded with a Na^+^ ion indicator, Na^+^ benzofuran isophthalate (SBFI, 5 μM) (Ion Biosciences) with Pluronic F-127 and probenecid for 2 hours in PBS with calcium and magnesium. Cells were then washed twice at room temperature using K^+^ free HEPES-containing salt solution (in mM: 20 HEPES, 130 NaCl, 1 CaCl_2_, 1 MgCl_2_, with pH 7.4 adjusted with NaOH) and incubated at room temperature for 1 hour. Directly before imaging, the solution was replaced with a solution containing 0, 3, 9 mM K^+^ (obtained by adding KCl from a 9 M stock to the solution) or by Tyrode’s solution (in mM: 137 NaCl, 5.4 KCl, 0.3 NaH_2_PO_4_, 1 MgCl_2_, 2 CaCl_2_, 10 glucose, 10 HEPES, with pH 7.4). Fluorescence was recorded using a FlexStation 3 plate reader. The excitation wavelengths were 340 nm and 380 nm and the emission filter was 510 nm. Fluorescence was recorded every minute for a period of 30–60 min. Data are displayed as the ratio of fluorescence recorded at 340 nm and 380 nm. Time zero is defined as the time at which fluorescence recordings are initiated. Total Na^+^ accumulation in the indicated assays in the manuscript (24 or 48 hours) were taken at time zero before K^+^ was added to initiate the flux assay.

Digitoxin was prepared as a 10 M stock solution in DMSO and diluted to a final concentration of 10 μM in HEPES or Tyrode’s recording solution. Monensin and gramicidin were used at a final concentration of 10 μM. Control samples contained only vehicle (0.1% DMSO).

### Metabolomics

U-^13^C-glucose tracing experiments, *sgLuc* and *sgFth1* cells were seeded into 6 well plates in either untreated or FAC treated conditions in DMEM with 10%FBS and 1%P/S. The cells were then grown for either 24 hours or 56 hours in under those treatment conditions. Prior to collection, cells were washed with 0.9% saline and media replaced with DMEM glucose tracing media which contained 25 mM U-^13^C-glucose, 4 mM glutamine, and dialyzed FBS. The cells were grown in this media for 20 min at 37°C. The cells were then washed and collected on ice with 0.9% saline, gently spun down in conical tubes, and flash frozen. For LC-MS, cell pellets were submitted to the NYULMC Metabolomics Laboratory. Briefly, metabolites were extracted and resolved by liquid chromatography using a p-HILIC column and peak heights were analyzed using Xcalibur (ThermoFisher Scientific). All metabolites were normalized to the closest labelled amino acid standard and also the respective cell number of the sample.

### Immunohistochemistry

Tumors were fixed in 10% formalin overnight and embedded in paraffin. Hematoxylin and eosin and immunohistochemical analysis was performed as described (*39*). In brief, slides were deparaffinized in xylene and rehydrated sequentially in ethanol. Antibody retrieval was performed by incubating slides in sodium citrate buffer (pH 6) in a pressure cooker for 15 min. Slides were quenched in hydrogen peroxide (3%) and blocked in normal serum for 1 h at room temperature. Primary antibody was applied overnight at 4°C then developed using vectastain Elite ABC kit (Vector labs PK-6100) and DAB (Vector labs SK-4100). Slides were counterstained with hematoxylin (Vector Labs H-3401). CD8a (eBioscience, 14–0808-82) was used at a 1:100 dilution. For CD8a^+^ staining, the percentage of CD8 positive cells in a defined tumor area were calculated using ImageJ and represented as a percentage of total tumor area.

### Immunoblotting

Immunoblotting was performed as previously described. Briefly, whole cell lysates were generated in modified radioimmunoprecipitation (RIPA) buffer (50 mM Tris-HCl pH 8.0, 150 mM NaCl, 2 mM EDTA, 1% NP-40, and 0.1% SDS, without sodium deoxycholate supplemented with protease (ThermoFisher,) and phosphatase (10 mM NaF, 1 mM Na3VO4, 10 mM β-glycerophosphate, and 10 mM sodium pyrophosphate) inhibitors. Protein lysates (25 μg) were loaded into 4%–20% gradient gels (BioRad, 4561096) and transferred to nitrocellulose (Thermo, 88018) or PVDF (BioRad 1620177) membranes with 1X transfer buffer (Tris-glycine) and 10% methanol. Membranes were blocked with 5% bovine serum albumin (Sigma, A2058) for 1 hour at room temperature before incubation overnight at 4°C with primary antibody. The following day membranes were washed in TBST and incubated for 1 hour with appropriate horseradish peroxidase-conjugated secondary antibody (1:5000) for 1 hour. Membranes were stripped with mild stripping buffer (15 g glycine, 1 g SDS, 10 ml Tween20, pH 2.2, for 1 liter of stripping buffer). Membranes were visualized by chemiluminescence (Bio-rad, 1705061) using a ChemiDoc (Bio-Rad). For LKB1 protein stability assays, *sgFth1* cells either untreated or treated with 100 μM of FAC for 48 hours. The cells were then subject to treatment with CQ (lysosomal inhibitor, 20 μM) or bortezomib (proteasomal inhibitor, 100 nm) for 5 hours and probed for LKB1 and ERK II.

Antibodies used for western blotting are: rabbit anti-FTH1 (Cell Signaling, catalog #4393, clone D1D4, RRID: AB_11217441; 1:1000, overnight at 4°C), mouse anti-ERK2 (Santa Cruz, catalog #sc-1647, clone D-2, RRID: AB_627547; 1:1000, overnight at 4°C), mouse anti-ATP1A1 (Santa Cruz, catalog #sc-21712, clone C464.6, RRID: AB_626713; 1:500, overnight at 4°C), mouse anti-SMG1 total (Santa Cruz, catalog #sc-374557, clone E-4, RRID: AB_11008478; 1:1000, overnight at 4°C), rabbit anti-phospho-SMG1 Thr3550 generated in this manuscript (catalog #N/A, clone N/A, RRID: this study; 1:100, overnight at 4°C), rabbit anti-LKB1 (Cell Signaling, catalog #3047, clone D60C5, RRID: AB_2198327; 1:1000, overnight at 4°C), rabbit monoclonal anti-phospho-HDAC4 Ser246/HDAC5 Ser259/HDAC7 Ser155 (Cell Signaling, catalog #3443, clone N/A, RRID: AB_2118723; 1:1000, overnight at 4°C), anti-SIK1 phospho-T182/SIK2 phospho-T175/SIK3 phospho-T163 antibody [EPR19196] (Abcam, catalog #ab199474, clone EPR19196, RRID: AB_3674042; 1:500, overnight at 4°C), rabbit anti-phospho-PDH S273 (Cell Signaling, catalog #31866, clone N/A, RRID: AB_2799014; 1:1000, overnight at 4°C), rabbit anti-PDH total (Cell Signaling, catalog #2784, clone N/A, RRID: AB_2162928; 1:500, overnight at 4°C), rabbit anti-phospho-UPF1 S1107 (Cell Signaling, catalog #84283, clone N/A, RRID: discontinued; 1:1000, overnight at 4°C), rabbit anti-UPF1 (Cell Signaling, catalog #12040, clone D15G6, RRID: AB_2797806; 1:1000, overnight at 4°C), rabbit anti-SLC8A2 (Thermo, catalog #PA5–77563, clone N/A, RRID: AB_2736625; 1:200, overnight at 4°C), rabbit anti-SLC16A3 (Cell Signaling, catalog #81569, clone F2K6A, RRID: AB_3676285; 1:1000, overnight at 4°C), mouse anti-p62 (Abnova, catalog #H00008878-M01, clone 2C11, RRID: AB_437085; 1:1000, 1 hour. at room temperature), rabbit anti-ubiquitin (Cell Signaling, catalog #58395, clone N/A, RRID: AB_3075532; 1:1000, overnight at 4°C), rabbit anti-IL36G (Abclonal, catalog #A10165, clone N/A, RRID: AB_2757692; 1:200, overnight at 4°C), anti-rabbit IgG HRP-linked antibody (Cell Signaling, catalog #7076S, clone N/A, RRID: AB_330924; 1:10,000, overnight at 4°C), and anti-rabbit IgG HRP-linked antibody (Cell Signaling, catalog #7074S, clone N/A, RRID: AB_2099233; 1:10,000, overnight at 4°C).

### RNA sequencing

RNA isolation was performed on 2D cells using TRizol (Thermo, 15596026) and PureLink RNA Midi Kit (Thermo, 12183025) according to the manufacturer’s instructions. Briefly, 10 cm plates of *sgLuc* or *sgFth1* cells were either untreated or treated with FAC for 48 hours prior to collection. Bulk RNA-seq analysis was performed by Novogene using DESeq2 (*47*). The volcano plot was generated with a *P* value cutoff of 0.001 for significance and a threshold of 3.5 log2 fold-change. Presentation of transcript data throughout the manuscript uses FPKM values unless otherwise stated.

### Electron microscopy

The electron microscopy was performed as previously described. Briefly, HY19636 cells with *sgLuc* or *shFth1* were plated in 10 cm plates and either untreated or treated with FAC for 48 hours. The cells were then fixed using 0.1 M sodium cacodylate buffer (pH 7.4) comprising 2.5% glutaraldehyde and 2% paraformaldehyde for overnight at 4°C. The cells were then stained with 1% osmium tetroxide and 1% potassium ferrocyanide for 1 hour at 4°C after which block staining was performed in 0.25% aqueous uranyl acetate for overnight at 4°C. The samples were then processed an cut into 70 nm sections and mounted on copper grids. A total of 10 images per condition were taken and used for subsequent quantification. The image analysis was performed in a blinded manner from on all mitochondria present in the cell. Quantification of TEM data, image processing, and analysis was done using ImageJ. Mitochondrial quality was determined by the percentage of mitochondria in sample that had a defined outer mitochondrial border and easily identifiable cristae structures.

### Calcium imaging

To assess cytosolic calcium stable cells lines expressing GCaMP6s reporter were generated by infecting HY19636 cells with GCamp6s lentivirus (Addgene plasmid, #164591) and then sorted for median fluorescence using fluorescence assisted cell sorting (FACS). The cells containing *sgLuc* or *sgFth1* constructs were then seeded at density of 4,000 cells per well into black-walled 96 well plates in either untreated or FAC treated conditions. The cells were grown for 48 hours in these conditions before they were washed with PBS and the media replaced with Fluobrite media. The cells were then assayed on the Agilent Cytation C10. The total fluorescence and cell number was captured at 10x magnification and the data reported as the total fluorescence divided by cell number for a particular condition.

For calcium imaging with treatments, *sgFth1* + GCaMP6s reporter cells were either treated with vehicle (DMSO), 20 μM chloroquine, or 100 nm Bortezomib for 5 hours. The cells were washed with PBS and then imaged in Fluobrite media immediately following treatment. The cells were then assayed on the Agilent Cytation C10. Representative images of the treated conditions are presented in Supplemental 5.

### Antibody production

Custom phosphor-SMG1 Threonine 3550 was generated by Yenzym Antibody services. Briefly, the antibody was generated by immunizing rabbits against VRSNTGQK-**pT**-QPDV. The resulting serum was then affinity purified and utilized in immunoblots to detect phosphorylation of SMG1.

### Quantitative reverse-transcriptase PCR

qRT-PCR was peformed as described previously (*38*). Briefly, total RNA was extracted from cells in 2D culture using TRIzol (Thermo, 15596026) and PureLink RNA Mini Kit (Thermo, 12183025). Cells were grown in 6-well plates and either untreated or treated with FAC for 48 hours prior to collection. For inhibitor experiments, cells were grown in vehicle or treatment conditions for 24 or 48 hours prior to collection. Reverse transcription was performed using Superscript Vilo IV (Thermo, 11766050) with oligo-dT primers. Quantitative PCR was performed with SYBR Green Supermix (Bio-rad, 1725274) on the CFX96 real-time PCR machine (Bio-Rad). The quantity of mRNA was calculated using ΔCT method normalizing to 18S.

Inhibitors:

SIKi (HG-9-91-01) – Cayman Chemicals 19179

KVS0001 – MedChemExpress HY-161111

Ionomycin – Thermo -I24222

HDACi – MedChemExpress CHDI-390576

Primer Sequences are as follows:

Il36g F 5′-TCCTGACTTTGGGGAGGTTTT-3′

Il36g R 5′-TCACGCTGACTGGGGTTACT-3′

Hmox F 5′-GAGCCTGAATCGAGCAGAAC-3′

Hmox R 5′-AGCCTTCTCTGGACACCTGA-3′

Nqo1 F 5′-AGCGTTCGGTATTACGATCC-3′

Nqo1 R 5′-AGTACAATCAGGGCTCTTCTCG-3′

Ddit3 F 5′-AAGCCTGGTATGAGGATCTGC-3′

Ddit3 R 5′-TTCCTGGGGATGAGATATAGGTG-3′

18S F 5′-GTAACCCGTTGAACCCCATT-3′

!8S R 5′-CCATCCAATCGGTAGTAGCG-3′

Human:

il36g human_F1 5′-AGGAAGGGCCGTCTATCAATC-3′

il36g human_R1 5′-CACTGTCACTTCGTGGAACTG-3′

### RNA stability assays

Total RNA was extracted from cells in 2D culture using TRIzol (Thermo, 15596026) and PureLink RNA Mini Kit (Thermo, 12183025). For *sgFth1*, cells were grown in 6-well plates and either untreated or treated with FAC for 48 hours prior to collection. Cells were then treated with actinomycin D (10 μg/ml) at 3 hours and 6 hours before collection or DMSO for collection at time zero. Reverse transcription was performed using Superscript Vilo IV (Thermo, 11766050) with oligo-dT primers. Quantitative PCR was performed with SYBR Green Supermix (Bio-rad, 1725274) on the CFX96 real-time PCR machine (Bio-Rad). The quantity of mRNA was calculated using ΔCT method normalizing to 18S. For NMD inhibitor experiments, *sgLuc* cells were treated for 24 hours with KVS0001 (.5 μM, MedChemExpress HY-161111). Cells were then treated with actinomycin D (10 μg/ml) at 3 hours and 6 hours before collection or DMSO for collection at time zero. Reverse transcription was performed using Superscript Vilo IV (Thermo, 11766050) with oligo-dT primers. Quantitative PCR was performed with SYBR Green Supermix (Bio-rad, 1725274) on the CFX96 real-time PCR machine (Bio-Rad).

### pHrodo green assays

pHrodo green (Thermo, P35373) is a fluorescent dye that detects cytoplasmic pH. Increased fluorescence is indicative of acidified intracellular environment whereas decreased fluorescence is indicative of alkalinization. Cells were cultured in 96-well white-walled plates at 37°C until they reached 80–90% confluence. Cells were either pre-treated with 100uM FAC for 48 hours prior to the assay or FAC was added via the Flexstation after time zero measurement. The pH-Rodo green dye was added as per manufacturer’s instructions. Briefly, 10 μl of pHrodo green was added to 100 μl of Powerload concentrate. This solution was added to Fluobrite media. Cells in the 96 well plate were washed once and the staining solution in Fluobrite media was added to the plate. The cells were stained in the dark for 30 minutes at room temperature. The cells were then imaged on Spectrmax3 or Flexstation with 509/533 excitation emission.

### ICP-MS for tissue iron content

Inductively-coupled plasma-mass spectrometry (ICP-MS) was used to quantify tissue iron content of pancreas, liver, and lung. To do this, pancreas, liver, and lung were collected from 8–10 week old C57BL/6 animals and flash frozen. These tissues were shipped on dry ice to Utah Iron and Heme Core Facility which performed the tissue ICP-MS. For the perfusion, mice were anesthetized and perfused 10 ml of 0.9% saline through the heart prior to collection. Tissues were collected after perfusion, flash frozen, and shipped on dry ice to the Utah Core.

### Flow cytometry

To assess cell death, we utilized flow cytometry and DAPI to stain for dead cells. Both *sgLuc* and *sgFth1* cells were seeded in 6-well plates and either untreated or treated with FAC for 48 hours. The cell supernatant was collected and cells were harvested by gentle trypsinization. Complete media was added to trypsinized cells and the cells and supernatant were spun down and washed with PBS. The cells were then stained in FACS buffer (HBSS, 1 mM EDTA, 1% BSA) with DAPI (2 μg/ml) for 2 min. As a positive control for DAPI staining, cells were treated with Bortezomib (100 nm) for 24 hours prior to collection. DAPI stained cells were then washed once in FACS buffer, resuspended in FACS buffer, and assessed by ZE5 flow cytometer.

### LKB1 cDNA foot-printing

The 5′UTR fragment of *Lkb1* and firefly luciferase (fLuc) was purchased from TWIST biosciences containing a T7 promoter and a strong transcriptional terminator sequence.

#### Lkb1

CAATCCGCCCTCACTACAACCGTAATACGACTCACTATAGGGCGCGGGGCCCAAGTGGCGAACATGACCTAGCGGCCCGCGCGCCGCGACGGCGGACCGTCGCCTCCGTCGGAAGCAGCGTCCCCGGGGACCCGAATTGGGGGGACGCGAGGGTTGGGGGGGCTCATTGCTTTTTTTTTTTTCATTCTTATTTTCATTTTTTTTCTCCCTGAGCACCTAGAA-GAAAAGGGGAAAATCAAAAGTGAAGAATTGGCGCCCAGGAAGCGGACGTGGACCCGGTTGTGGGGACCGGGAGAGTTGTGGAGGTCGTTCCTGTTTTTCCCCGTTCCTTTT-TTCCCCTTCTTGGAACATTTGGAAGAAGCGGG-TGGGGGGGGGGGAATTCGAACTTGAAAAGAATTGGCGCTCCCGAAGGGGACGAGGACAAAGAGTGGGCCAGGATGGACGTGGCGGACCCCGAGCCGTTGGGCCTTTTCTCCGAGGGCGAGCTGATGTCGGTGGGCATGGACACCTTCATCCACCGCATCGACTCCACCGAGGTAATCTACCAGCCGCGCCGCAAACGCGCCAAGCTCATCGGCAAGTACCTGATGGGGGACCTGCTCGGGGAGGGCTCGTACGGCAAGGTGAAGGAGGTGCTGGACTCCGAGACCTTATGCCGCAGGGCGGTCAAGATCCTCAAGAAGAAAAAGCTGCGCAGGATCCCCAATGGAGAGGCCAACGTCAAGACCGCTGAGCAATAACTAGCCTACTCTGGCGTCGATGAGGGA

#### fLuc

TAATACGACTCACTATAGGATGGAAGATGCCAAAAACATTAAGAAAGGCCCAGCGCCATTCTACCCACTCGAAGACGGGACCGCTGGCGAGCAGCTGCATAAAGCCATGAAGCGCTACGCCCTGGTGCCCGGCACCATCGCCTTTACCGACGC-ACATATCGAGGTGGACATTACCTACGCCGAGTACTTCGAGATGAGCGTTCGGCTGGCAGAAGCTATGAAGCGCTATGGGCTGAATACAAACCATCGGATCGTGGTGTGTAGCGAGAATAGCTTGCAGTTCTTCATGCCCGTGTTGGGTGCCCTGTTCATCGGTGTGGCTGTGGCCCCAGCTAACGACATCTACAACGAGCGCGAGCTGCTGAACAGCATGGGCATCAGCCAGCCCACCGTCGTATTCGTGAGCAAGAAAGGGC-TGCAAAAGATCCTCAACGTGCAAAAGAAGCTACCGA-TCATACAAAAGATCATCATCATGGATAGCAAGACCGACTA-CCAGGGCTTCCAAAGCATGTACACCTTCGTGACTTCCCATTTGCCACCCGGCTTCAACGAGTACGACTTCGTGCCCGAGAGCTTCGACCGGGACAAAACCATCGCCCTGATCATGAACAGTAGTGGCAGTACCGGATTGCCCAAGGGCGTAGCCCTACCGCACCGCACCGCTTGTGTCCGATTCAGTCATGCCCGCGACCCCATCTTCGGCAACCAGATCATCCCCGACACCGCTATCCTGAGCGTGGTGCCATTTCACCACGGCTTCGGCATGTTCACCACGCTGGGCTACTTGATCTGCGGCTTTCGGGTCGTGCTCATGTACCGCTTCGAGGAAGAGCTATTCTTGCGCAGCTTGCAAGACTATAAGATTCAATCTGCCCTGCTGGTGCCCACACTATTTAGCTTCTTCGCTAAGAGCACTCTCATCGACAAGTACGACCTAAGCAACTTGCACGAGATCGCCAGCGGCGGGGCGCCGCTCAGCAAGGAGGTAGGTGAGGCCGTGGCCAAACGCTTCCACCTACCAGGCATCCGCCAGGGCTACGGCCTGACAGAAACAACCAGCGCCATTCTGATCACCCCCGAAGGGGACGACAAGCCTGGCGCAGTAGGCAAGGTGGTGCCCTTCTTCGAGGCTAAGGTGGTGGACTTGGACACCGGTAAGACACTGGGTGTGAACCAGCGCGGCGAGCTGTGCGTCCGTGGCCCCATGATCATGAGCGGCTACGTTAACAACCCCGAGGCTACAAACGCTCTCATCGACAAGGACGGCTGGCTGCATAGCGGCGACATCGCCTACTGGGACGAGGACGAGCACTTCTTCATCGTGGACCGGCTGAAGAGCCTGATCAAATACAAGGGCTACCAGGTAGCCCCAGCCGAACTGGAGAGCATCCTGCTGCAACACCCCAACATCTTCGACGCCGGGGTCGCCGGCCTGCCCGACGACGATGCCGGCGAGCTGCCCGCCGCAGTCGTCGTGCTGGAACACGGTAAAACCATGACCGAGAAGGAGATCGTGGACTATGTGGCCAGCCAGGTTACAACCGCCAAGAAGCTGCGCGGTGGTGTTGTGTTCGTGGACGAGGTGCCTAAAGGACTGACCGGCAAGTTGGACGCCCGCAAGATCCGCGAGATTCTCATTAAGGCCAAGAAGGGCGGCAAGATCGCCGTCTAG

In vitro transcription of mRNA from the DNA template was performed as per the manufacturer instructions (T7 Hi-Scribe, NEB, E2040S). The mRNA from the in vitro transcription as purified and used for downstream applications. To perform the foot-printing assay, primers were designed at the 3′ end of the *Lkb1* 5′UTR. The mRNA template was added to PCR tubes with either control buffer (100 mM Tris-HCl, 10 mM LiCl, 1 mM dNTP, 5 mM MgCl_2_, 5 mM DTT) or quadruplex inducing buffer (100 mM Tris-HCl, 10 mM KCl, 1 mM dNTP, 5 mM MgCl_2_, 5 mM DTT). Then 1 μM of LKB1 specific primer was added to the reaction. The conditions were then incubated at 65C for 5 min and allowed to gradually cool to room temperature. Then 1 μl of Superscript III reverse transcriptase () was added to the 20 μl reaction and put on a thermocycler for at 25°C for 10 min, 50°C for 50 min, and 85C for 5 min.

We then added 1.1 μl of 1 N NaOH to the 20 μL reaction and heated the sample at 70°C for 12 minutes to hydrolyze any remaining RNA. This was followed by the addition of 1.1 μl of 1 N HCl to neutralize the reaction. Following neutralization, 2x RNA dye was added to each sample and the sample heated at 85 for 2 minutes. Samples were cooled on ice prior to running on a denaturing urea gel. Samples were stained with SYBR gold for 10 minutes and visualized on iBright.

Lkb1_Reverse Primer: 5′-TCTTGACGTTGGCCTCTCCATTG-3′

fLuc_Reverse Primer: 5′-GCGATCTTGCCGCCCTTC-3′

### Antibody production

Custom phosphor-SMG1 Threonine 3550 was generated by Yenzym Antibody services. Briefly, the antibody was generated by immunizing rabbits against VRSNTGQK-**pT**-QPDV. The resulting serum was then affinity purified and utilized in immunoblots to detect phosphorylation of SMG1.

### Identification of quadruplex and in vitro transcription and purification of RNA

QGRS Mapper was used to predict RNA G-Quadruplexes present in either *Lkb1* or *fLuc*. Hairpins used for G-quadruplex characterization were transcribed in vitro with T7 RNA polymerase from DNA templates encoding for the RNA and T7 promoter [Integrated DNA Technologies (IDT)]. The transcription reaction consisted of 0.64 μM DNA template annealed to T7 promoter primer (5′-TAATACGACTCACTATAGGG-3′, IDT), 8 mM of each rNTP, 10% polyethylene glycol 8000, 20 mM MgCl2, 1 × transcription buffer (5 mM Tris, pH 8, 5 mM spermidine, 10 mM DTT), and in-house expressed and purified T7 bacterial polymerase. RNA was in vitro transcribed at 37°C for 3 hours, then purified by high-performance liquid chromatography (HPLC) on a DNAPacTM PA200 column (Thermo Scientific) using 12.5 mM Tris HCl and 6 M urea and eluted with a gradient of 0 to 500 mM sodium perchlorate. RNA was then desalted using Sep-Pak Plus Long C18 columns (Waters). Samples were then separated into aliquots for either chemical probing experiments, circular dichroism (CD) spectroscopy, or nuclear magnetic resonance (NMR) spectroscopy. Samples were lyophilized and resuspended in the appropriate buffers to carry out experiments described below.

### Chemical probing

Chemical probing was performed as described previously (*48*), (*49*). Briefly, in vitro transcribed RNA was heated to 95°C for 5 min, then immediately cooled on ice. RNA was refolded at 37°C in a buffer containing 100 mM HEPES (pH 8), 10 mM MgCl2, and 100 mM of NaCl, LiCl, or KCl. For DMS-MaP experiments, RNA was probed at varying concentrations to confirm single hit kinetics (*50*, *51*) settling on 10.1 mM dimethyl sulfate at 37°C for 15 min and quenched with 333 mM dithiothreitol. For SHAPE-MaP experiments, RNA was probed at varying concentrations of 5-Nitroisatoic Anhydride (5NIA), settling on 3 mM or 6 mM depending on refolding buffer. RNA was incubated with 5NIA for 25 min at 25°C and quenched with 333 mM dithiothreitol. All RNA samples were cleaned up with RNAClean XP beads. DMS samples were reverse transcribed using Induro Reverse Transcriptase (New England Biolabs, M0681L), 5NIA samples were reverse transcribed using SuperScript II Reverse Transcriptase (Invitrogen, 18064071). The resulting cDNA was purified using RNAClean XP beads, amplified using PrimeSTAR GXL DNA polymerase (Takara Bio, R050A), and cleaned using AMPureXP beads (Beckman, A63881)

Primers used for cDNA amplification:

LKB1_Forward Primer5′-GCGCGGGGCCCAAGTGGC-3′

LKB1_Reverse Primer5′-TCTTGACGTTGGCCTCTCCATTG-3′

G-quadruplex hairpins

Hairpin construct 1ggCCCCGUUCCUUUUUUCCCCUUCUUAAGAAGCGGGUGGGGGGGGGGGcc

Hairpin construct 2ggUUUUUCCCCGUUCCUUUUUUAAGAAGCGGGUGGGGGGGGGcc

Sequencing was performed using Oxford Nanopore sequencing Native Barcoding Kit (Oxford Nanopore Technologies, SQK-NBD114.24) and sequenced using either MinION (FLO-MIN114) or Flongle (FLO-FLG114) flow cell as per manufacturer’s instructions. Basecalling was performed using the Dorado high-accuracy model and aligned with Bowtie2, then analyzed using Shapemapper2. The RNA was folded in RNAStructure Fold with SHAPE constraints to determine the minimum free energy structure. Changes in reactivity were determined using deltaSHAPE. RNA secondary structure was made using RNAcanvas (*48*, *52*–*58*).

### Circular dichroism

CD spectra were measured using a Jasco J-1500 CD spectrometer with wavelengths scanning from 320 nm to 200 nm. Spectra were recorded using a 1 mm path length quartz cuvette with RNA concentrations ranging between 5–6 μM. Samples were dissolved in a buffer containing 10 mM MgCl2 and 15 mM sodium phosphate (NaP) at pH 6.5. These buffers were supplemented with either 150 mM KCl, NaCl, or LiCl. CD spectra were measured in varying folding conditions. RNA was either thawed from frozen and immediately measured, heated at 95°C for 5 minutes and slowly cooled to room temperature on the benchtop, or heated at 95°C for 5 minutes and immediately placed on ice. Scans were performed at 25°C and each final spectrum is the average of three accumulation trials taken with a step size of 1 nm, a time per point of 1 s, and a bandwidth of 1 nm. A buffer only blank was subtracted from each spectrum. Ellipticity (deg) was normalized to molar ellipticity (θmolar, deg.·cm^2^·dmol^−1^) to account for varying RNA concentrations. The spectra for all hairpins have maxima centered around 267 nm and minima near 240 nm (*59*, *60*).

### Nuclear magnetic resonance spectroscopy

All NMR experiments were carried out using 55–68 μM unlabeled RNA for 1H 1D imino experiments with standard Bruker pulse programs on a Bruker 600 MHz Avance III spectrometer equipped with a TCI cryogenic probe at 298 K. Experiments were performed in 15 mM NaP pH 6.5, 10 mM MgCl2, 10% D2O, and either 150 mM KCl or 150 mM LiCl. 1H spectra were processed and analyzed using Bruker TopSpin (4.4) and CCPN (3.3.2.3) (*61*–*63*).
