# Supplementary material for: Iron overload suppresses LKB1 and induces IL36G anti-tumor immunity in PDAC metastasis
Source: Sci Adv. 2026 Jul 17;12(29):eadz8681. doi: 10.1126/sciadv.adz8681 (PMC13378540; doi:10.1126/sciadv.adz8681)
Supplement: Supplementary file 1 — Figs. S1 to S7 Legend for data S1 [file sciadv.adz8681_sm.pdf]

Science Advances
AAAS

Supplementary Materials for

Iron overload suppresses LKB1 and induces IL36G anti-tumor immunity in PDAC metastasis

Douglas E. Biancur *et al.*

Corresponding author: Douglas E. Biancur, douglas.biancur@nyulangone.org;
Richard Possemato, richard.possemato@nyulangone.org

Sci. Adv. **12**, eadz8681 (2026)
DOI: 10.1126/sciadv.adz8681

The PDF file includes:

Figs. S1 to S7
Legend for data S1

Other Supplementary Material for this manuscript includes the following:

Data S1

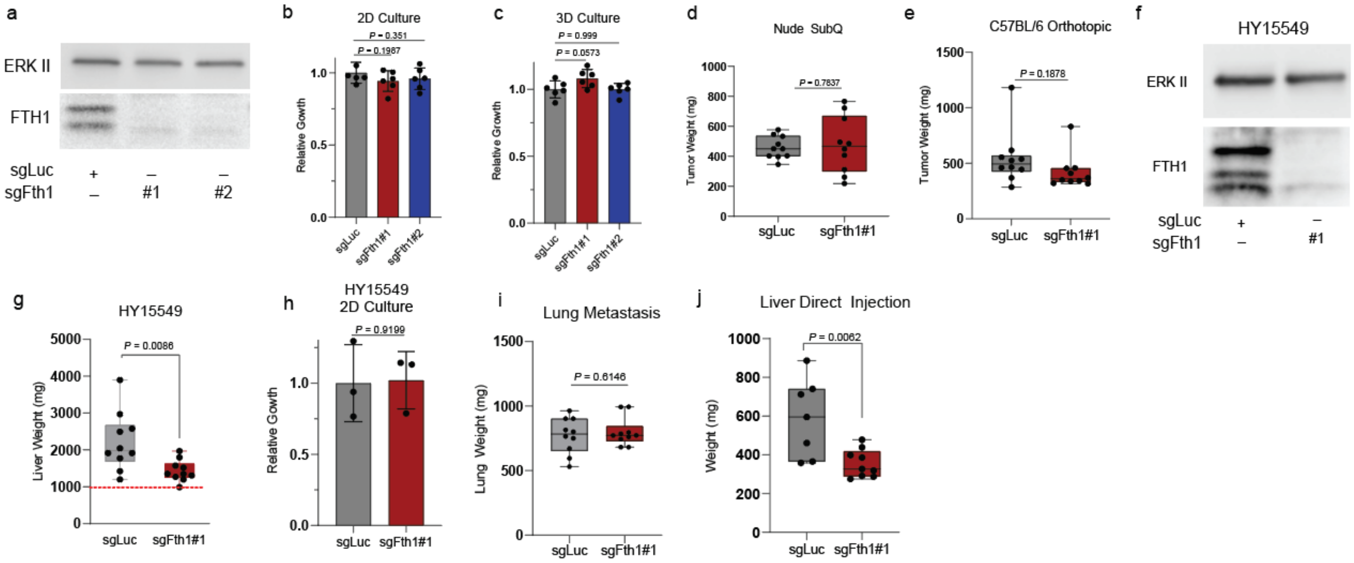

Supplemental Fig. 1: Validation of liver metastasis screen

a) Representative immunoblot of *sgLuc* and *sgFth1* (KO#1 and KO#2) probed for ERKII and FTH1 to demonstrate knockout in HY19636 cells. **b)** Relative proliferation rates of *sgLuc* and *sgFth1* cells in 2D culture to validate screening data in culture. Data are plotted as relative cell proliferation in arbitrary units. Data represents the mean and error bars depict ± SD of three independent wells from a representative experiment. **c)** Relative proliferation rates of *sgLuc* and *sgFth1* cells in 3D culture to validate screening data in culture. Data are plotted as relative cell proliferation normalized to day 0 in arbitrary units. Data represents the mean and error bars depict ± SD of six independent wells from a representative experiment. **d)** Implantation of *sgLuc* (n=10) and *sgFth1* (n=10) cells demonstrating no change in tumor burden between control and knockout cells in athymic nude subcutaneous tumors. Data represents the median and error bars depict the range of individual tumor weights. **e)** Implantation of *sgLuc* (n=10) and *sgFth1* (n=10) cells demonstrating no change in tumor burden between control and knockout cells in syngeneic C57BL/6 orthotopic tumors. Error bars depict the range of individual tumor weights. **f)** Representative immunoblot of *sgLuc* and *sgFth1* probed for ERKII and FTH1 to demonstrate knockout in HY15549 cells. **g)** Implantation of *sgLuc* (n=10) and *sgFth1* (n=10) cells demonstrating decreased tumor burden in knockout cells compared to controls in syngeneic C57BL/6 liver metastases in HY15549 cells. Data represents the median and error bars depict the range of individual tumor weights. **h)** Relative proliferation rates of *sgLuc* and *sgFth1* cells in 2D culture in HY15549 cells. Data are plotted as relative cell proliferation normalized to day 0 in arbitrary units. Data represents the mean and error bars depict ± SD of three independent wells from a representative experiment. **i)** Implantation of *sgLuc* (n=10) and *sgFth1* (n=10) cells demonstrating no change in tumor burden in knockout cells compared to controls in syngeneic C57/BL6 lung metastases in HY19636 cells. Data represents the median and error bars depict the range of individual tumor weights. **j)** Implantation of *sgLuc* (n=7) and *sgFth1* (n=9) cells demonstrating no change in tumor burden in knockout cells compared to controls in syngeneic C57/BL6 lung metastases in HY19636 cells. Data represents the median and error bars depict the range of individual tumor weights. Statistical analysis was performed using GraphPad Prism. Unpaired, two tailed Student's t-tests were performed when comparing two groups to each other.

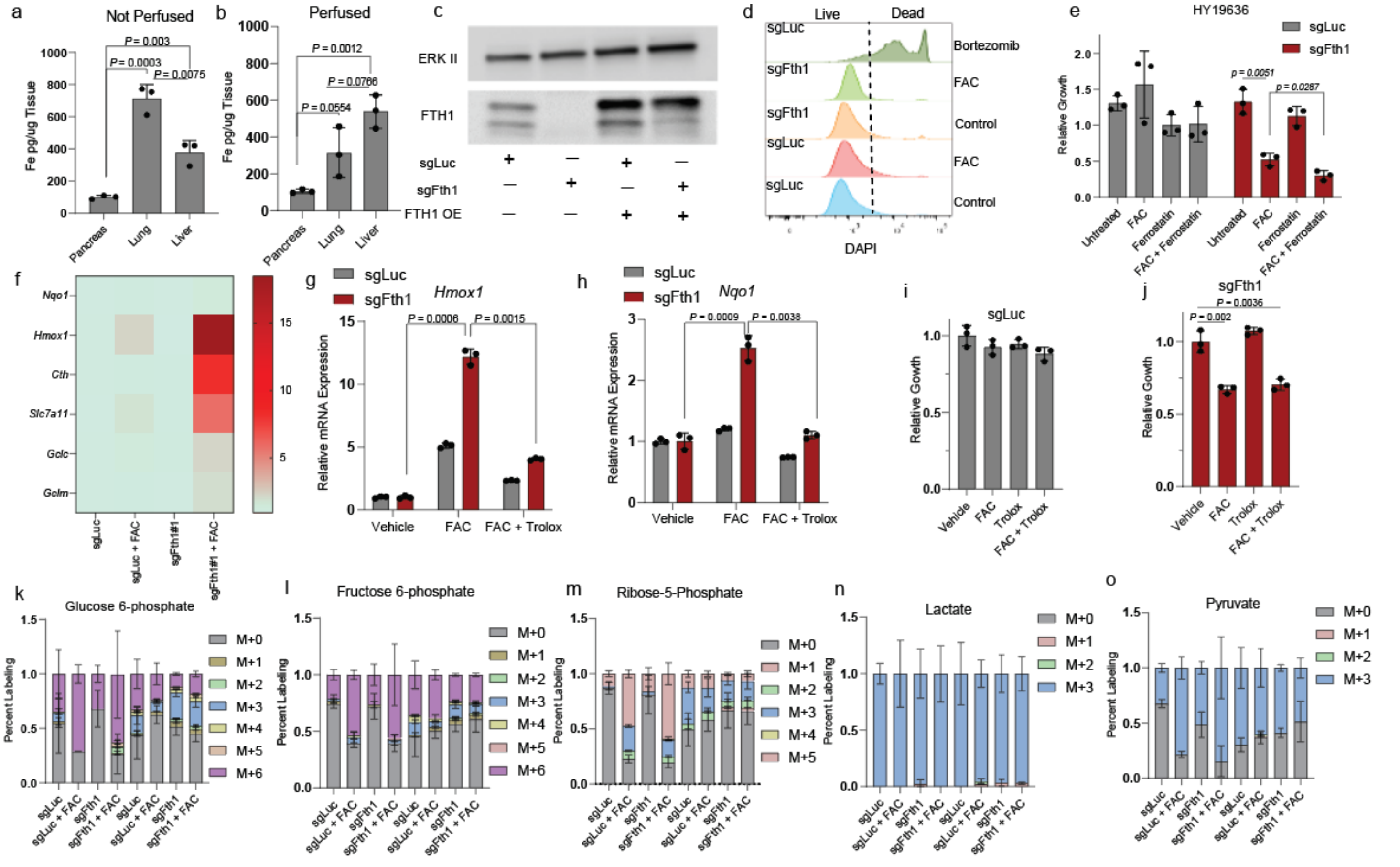

Supplemental Fig. 2: Iron overloaded cells reveal REDOX imbalance and altered Glycolysis

a) Quantification of tissue iron by ICP-MS of pancreas, lung, and liver in C57/BL6 mice. Data represents the mean and error bars represent ± SD of three individual tissues from individual mice. **b)** Quantification of tissue iron by ICP-MS of pancreas, lung, and liver in C57/BL6 mice after perfusion with normal saline. Data represents the mean and error bars represent ± SD of three individual tissues from individual mice. **c)** Representative immunoblot of *sgLuc* and *sgFth1* cells infected with either empty vector (EV) or *Fth1* cDNA (FTH1 OE) and probed for ERKII and FTH1 to demonstrate knockout and cDNA addback and probed for FTH1 and ERK II. **d)** Flow cytometry of *sgLuc* and *sgFth1* cells treated with 100uM of FAC for 48hrs and stained with DAPI to observe cell death. Treatment with bortezomib 100nM for 48hrs was used as a positive control for cell death. **e)** Relative proliferation rates of sgLuc and sgFth1 cells in untreated or FAC treated conditions (100uM FAC). Both sgLuc and sgFth1 cells were also treated with Ferrostatin (1uM) or the combination of FAC and Ferrostatin. The addition of Ferrostatin was unable to support the growth of sgFth1 cells under FAC treated conditions. Data are plotted as relative cell proliferation in arbitrary units. Data presented as mean and error bars depict ± SD of three independent wells from a representative experiment. **f)** Heat map of changes observed in antioxidant genes from RNA sequencing of *sgLuc* and *sgFth1* cells treated with FAC for 48hrs. **g)** RT-qPCR for HMOX in *sgLuc* and *sgFth1* cells untreated or treated with 100uM FAC and treated with either vehicle (DMSO) or Trolox (10uM) for 48hrs. Data represents the mean and error bars depict ± SD of three technical replicates from a representative experiment. **h)** RT-qPCR for NQO1 in *sgLuc* and *sgFth1* cells untreated or treated with 100uM FAC and treated with either vehicle (DMSO) or Trolox (10uM) for 48hrs. **j)** Relative proliferation rates of *sgLuc* cells in 2D culture treated with vehicle (DMSO) or Trolox (10uM). Data are plotted as relative cell proliferation normalized to day 0 in arbitrary units. Data represents the mean and error bars depict ± SD of three independent wells from a representative experiment. **k)** Relative proliferation rates of *sgFth1* cells in 2D culture treated with vehicle (DMSO) or Trolox (10uM). Data are plotted as relative cell proliferation in arbitrary units. Data represents the mean and error bars depict ± SD of three independent wells from a representative experiment. **k)** Glucose 6-phosphate percent labeling pattern stacked to 100% from U-13C-glucose tracing performed for 20 minutes in both sgLuc and sgFth1 cells treated with FAC at an early time point (24hrs) or a late time point (56hrs). Data presented as mean and error bars depict ± SD of three independent wells from a representative experiment. **l)**

Fructose 6-phosphate percent labeling pattern stacked to 100% from U-13C-glucose tracing performed for 20 minutes in both sgLuc and sgFth1 cells treated with FAC at an early time point (24hrs) or a late time point (56hrs). Data presented as mean and error bars depict ± SD of three independent wells from a representative experiment. **m)** Ribose 5-phosphate percent labeling pattern stacked to 100% from U-13C-glucose tracing performed for 20 minutes in both sgLuc and sgFth1 cells treated with FAC at an early time point (24hrs) or a late time point (56hrs). Data presented as mean and error bars depict ± SD of three independent wells from a representative experiment. **n)** Lactate labeling pattern stacked to 100% from U-13C-glucose tracing performed for 20 minutes in both sgLuc and sgFth1 cells treated with FAC at an early time point (24hrs) or a late time point (56hrs). Data presented as mean and error bars depict ± SD of three independent wells from a representative experiment. **o)** Pyruvate percent labeling pattern stacked to 100% from U-13C-glucose tracing performed for 20 minutes in both sgLuc and sgFth1 cells treated with FAC at an early time point (24hrs) or a late time point (56hrs). Data presented as mean and error bars depict ± SD of three independent wells from a representative experiment.

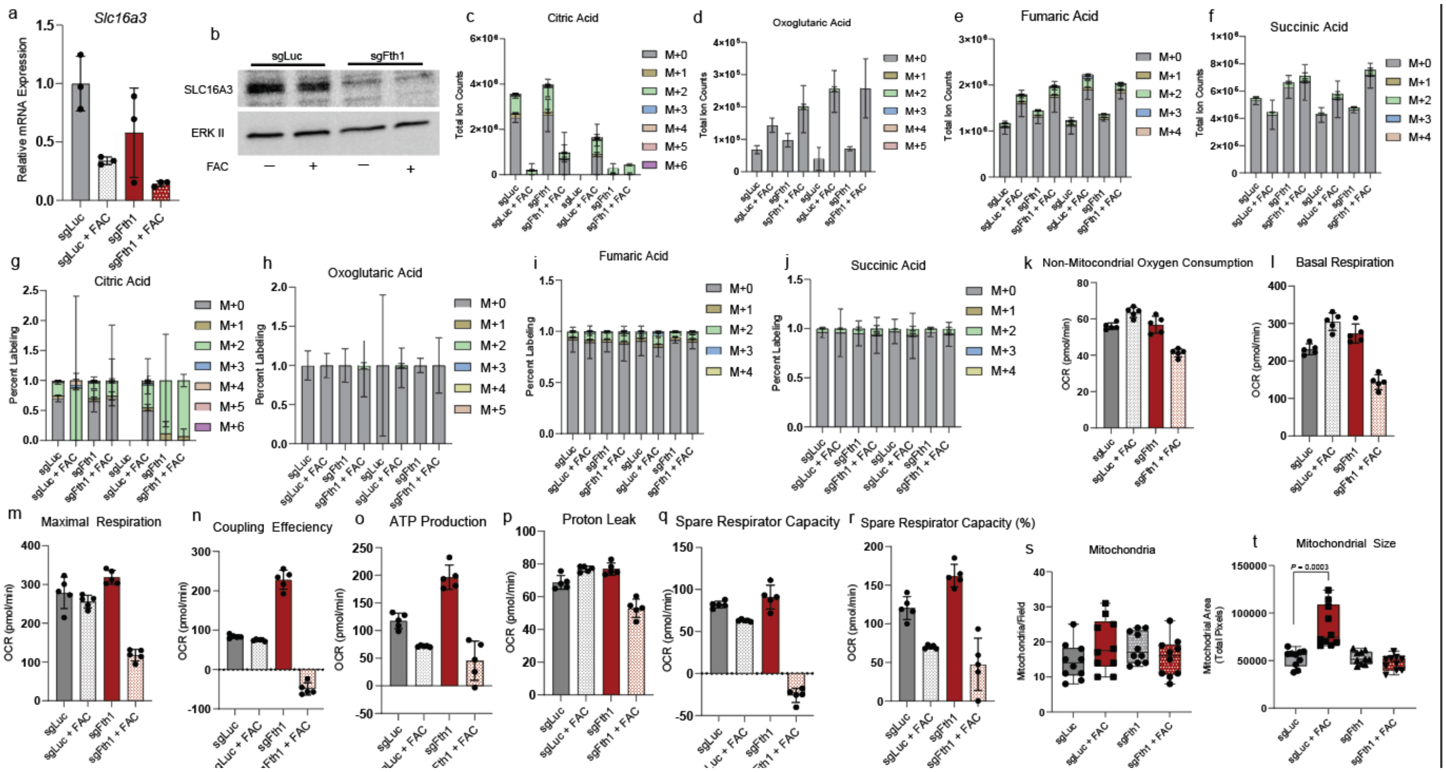

Supplemental Fig. 3: Iron overloaded cells reveal Mitochondrial Dysfunction

a) RNA sequencing for mRNA levels of SLC16A3 reveals transcriptional downregulation upon treatment with FAC in either *sgLuc* or *sgFth1* cells. Data represents the mean and error bars depict ± SD of three independent wells. **b)** Representative immunoblot of *sgLuc* and *sgFth1* cells untreated or treated with 100uM FAC for 48hrs and probed for ERKII and SLC16A3. **c)** Citric acid total ion current and labeling pattern from U-13C-glucose tracing performed for 20 minutes in both sgLuc and sgFth1 cells treated with FAC at an early time point (24hrs) or a late time point (56hrs). Data presented as mean and error bars depict ± SD of three independent wells from a representative experiment. **d)** Oxoglutaric acid total ion current and labeling pattern from U-13C-glucose tracing performed for 20 minutes in both sgLuc and sgFth1 cells treated with FAC at an early time point (24hrs) or a late time point (56hrs). Data presented as mean and error bars depict ± SD of three independent wells from a representative experiment. **e)**. Fumaric acid total ion current and labeling pattern from U-13C-glucose tracing performed for 20 minutes in both sgLuc and sgFth1 cells treated with FAC at an early time point (24hrs) or a late time point (56hrs). Data presented as mean and error bars depict ± SD of three independent wells from a representative experiment. **f)** Succinic acid total ion current and labeling pattern from U-13C-glucose tracing performed for 20 minutes in both sgLuc and sgFth1 cells treated with FAC at an early time point (24hrs) or a late time point (56hrs). Data presented as mean and error bars depict ± SD of three independent wells from a representative experiment. **g)** Citric acid percent labeling pattern stacked to 100% from U-13C-glucose tracing performed for 20 minutes in both sgLuc and sgFth1 cells treated with FAC at an early time point (24hrs) or a late time point (56hrs). Data presented as mean and error bars depict ± SD of three independent wells from a representative experiment. **h)** Oxoglutaric acid percent labeling pattern stacked to 100% from U-13C-glucose tracing performed for 20 minutes in both sgLuc and sgFth1 cells treated with FAC at an early time point (24hrs) or a late time point (56hrs). Data presented as mean and error bars depict ± SD of three independent wells from a representative experiment. **i)** Fumaric acid percent labeling pattern stacked to 100% from U-13C-glucose tracing performed for 20 minutes in both sgLuc and sgFth1 cells treated with FAC at an early time point (24hrs) or a late time point (56hrs). Data presented as mean and error bars depict ± SD of three independent wells from a representative experiment**.** **j)** Succinic acid percent labeling pattern stacked to 100% from U-13C-glucose tracing performed for 20 minutes in both sgLuc and sgFth1 cells treated with FAC at an early time point (24hrs)

or a late time point (56hrs). Data presented as mean and error bars depict ± SD of three independent wells from a representative experiment. **k)** Non-mitochondrial oxygen consumption rate of sgLuc and sgFth1 cells untreated or treated with FAC. Data are plotted as relative OCR to untreated sgLuc samples. Data presented as mean and error bars depict ± SD of five individual wells from a representative experiment. **l)** Basal respiration of sgLuc and sgFth1 cells untreated or treated with FAC. Data are plotted as relative OCR to untreated sgLuc samples. Data presented as mean and error bars depict ± SD of five individual wells from a representative experiment. **m)** Maximal respiration rate of sgLuc and sgFth1 cells untreated or treated with FAC. Data are plotted as relative OCR to untreated sgLuc samples. Data presented as mean and error bars depict ± SD of five individual wells from a representative experiment. **n)** Coupling efficiency of sgLuc and sgFth1 cells untreated or treated with FAC. Data are plotted as relative OCR to untreated sgLuc samples. Data presented as mean and error bars depict ± SD of five individual wells from a representative experiment. **o)** ATP production of sgLuc and sgFth1 cells untreated or treated with FAC. Data are plotted as relative OCR to untreated sgLuc samples. Data presented as mean and error bars depict ± SD of five individual wells from a representative experiment. **p)** Proton leak of sgLuc and sgFth1 cells untreated or treated with FAC. Data are plotted as relative OCR to untreated sgLuc samples. Data presented as mean and error bars depict ± SD of five individual wells from a representative experiment. **q)** Spare respirator capacity of sgLuc and sgFth1 cells untreated or treated with FAC. Data are plotted as relative OCR to untreated sgLuc samples. Data presented as mean and error bars depict ± SD of five individual wells from a representative experiment. **r)** Spare respirator capacity percentage of sgLuc and sgFth1 cells untreated or treated with FAC. Data are plotted as relative OCR to untreated sgLuc samples. Data presented as mean and error bars depict ± SD of five individual wells from a representative experiment**s)** Quantification of electron micrographs for total number of mitochondria per field. The analysis was performed with ImageJ analysis software. Data represents the median and error bars depict the range of ten independent micrographs **t)** Quantification of electron micrographs for mitochondrial size. Data represents the median and error bars depict the range of ten independent micrographs The analysis was performed with ImageJ analysis software. Error bars depict the range of ten independent micrographs. Statistical analysis was performed using GraphPad Prism. Unpaired, two tailed Student's t-tests were performed when comparing two groups to each other.

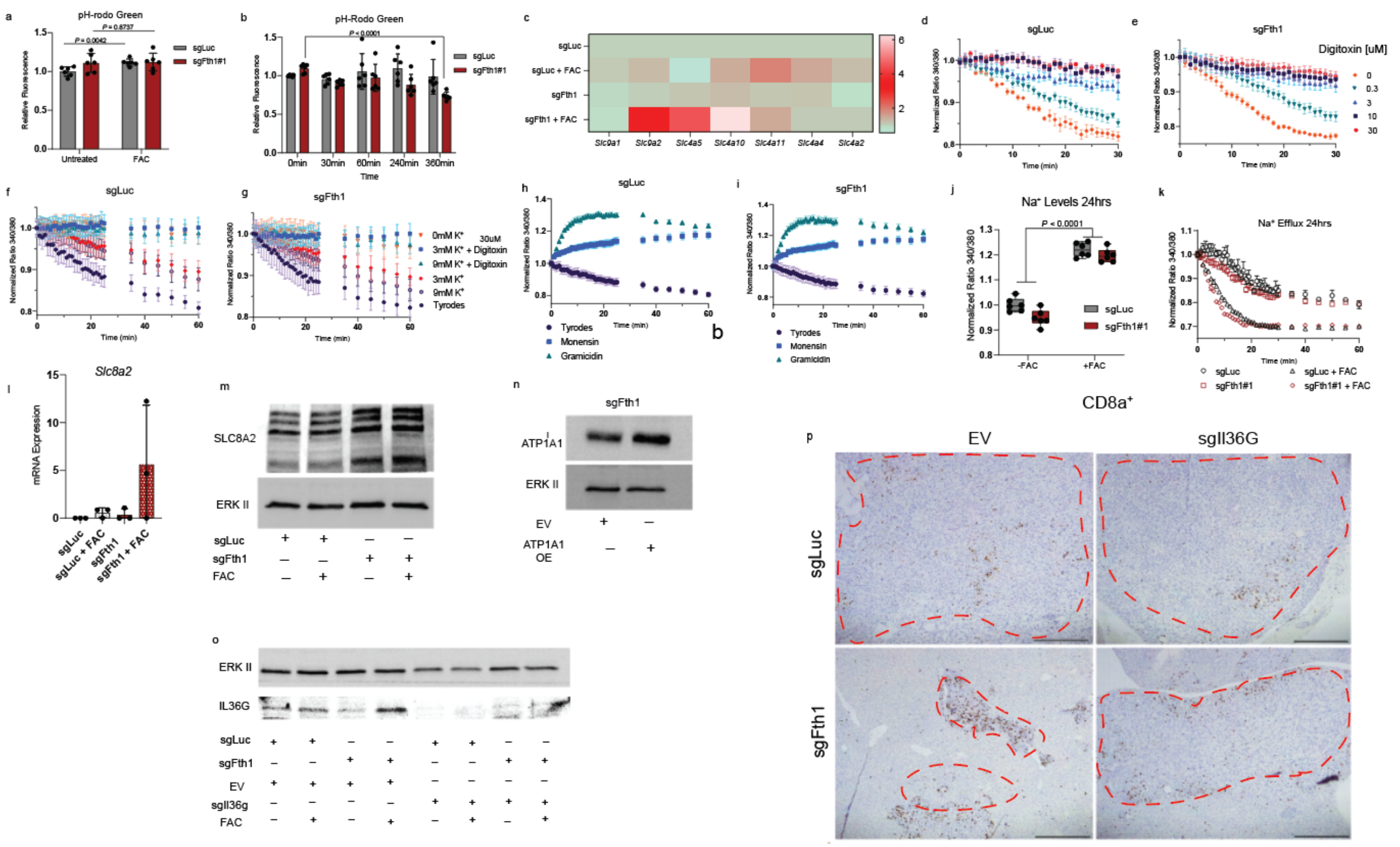

Supplemental Fig. 4 Assessing changes in ion homeostasis in response to iron

a) Relative fluorescence of pH dye pH-Rodo green in *sgLuc* and *sgFth1* cells either untreated or treated with 100uM FAC for 48hrs. Data are plotted as relative fluorescence normalized to *sgLuc* in arbitrary units. Data represents the median and error bars depict the range of ten independent micrographs error bars depict ± SD of six independent wells from a representative experiment. **b)** Relative fluorescence of pH dye pH-Rodo green in *sgLuc* and *sgFth1* cells either untreated or treated with 100uM FAC in a time course. Data are plotted as relative fluorescence normalized to *sgLuc* at time 0 in arbitrary units. Data represents the median and error bars depict the range of ten independent micrographs error bars depict ± SD of six independent wells from a representative experiment. **c)** Heat map of changes observed in pH regulating genes from RNA sequencing of *sgLuc* and *sgFth1* cells treated with FAC for 48hrs. **d)** Na^+/K^+-ATPase activity in *sgLuc* cells treated with increasing concentrations of digitoxin (0-30uM). Na^+ efflux as measured by SBFI fluorescence was used as a surrogate for Na^+/K^+-ATPase activity which was inhibited by digitoxin in a dose-dependent manner. Data represents the mean and error bars depict ± SEM of 6 independent wells from a representative experiment. **e)** Na^+/K^+-ATPase activity in *sgFth1* cells treated with increasing concentrations of digitoxin (0-30uM). Na^+ efflux was used as a surrogate for Na^+/K^+-ATPase activity which was inhibited by digitoxin in a dose-dependent manner. Data represents the mean and error bars depict ± SEM of 6 individual wells from a representative experiment. **f)** Na^+/K^+-ATPase activity in *sgLuc* cells treated with increasing concentrations of K^+ with and without digitoxin (30uM). Na^+ efflux was used as a surrogate for Na^+/K^+-ATPase activity which was inhibited by digitoxin in a dose-dependent manner. Error bars depict ± SEM of six independent wells from a representative experiment. **g)** Na^+/K^+-ATPase activity in *sgFth1* cells treated with increasing concentrations of K^+ with and without digitoxin (30uM). Na^+ efflux was used as a surrogate for Na^+/K^+-ATPase activity which was inhibited by digitoxin in a dose-dependent manner. Data represents the mean and error bars depict ± SEM of 6 individual well from a representative experiment. **h)** Total Na^+ accumulation was assessed in *sgLuc* cells with SBFI by incubating cells with either Tyrode's solution (control) or Na^+ ionophores gramicidin and monensin. Data represents the mean and error bars depict ± SEM of 6 individual well from a representative experiment. **i)** Total Na^+ accumulation was assessed in *sgFth1* cells with SBFI by incubating cells with either Tyrode's solution (control) or Na^+ ionophores gramicidin and monensin. Data represents the mean and error bars depict ± SEM of 6 individual well from a representative experiment. **j)** Total Na^+ levels in either *sgLuc* or *sgFth1* cells untreated or treated with 100uM FAC for 48hrs. Total Na^+ levels

are assessed with SBFI after incubating cells for 1hr in K^+ free buffer to prevent the activity of the Na^+/K^+-ATPase. Data presented as median and error bars represent the range of 6 individual wells from a representative experiment. **k)** Na^+/K^+-ATPase activity in *sgLuc* or *sgFth1* cells untreated or treated with 100uM FAC for 24 hrs. Na^+ efflux was used as a surrogate for Na^+/K^+-ATPase activity by reintroducing K^+ into the cell and monitoring decrease in SBFI fluorescence over time. Data presented as median and error bars represent ± SEM of six individual wells from a representative experiment. Na^+/K^+-ATPase activity in *sgLuc* or *sgFth1* cells untreated or treated with 100uM FAC for 48hrs. Na^+ efflux was used as a surrogate for Na^+/K^+-ATPase activity by reintroducing K^+ into the cell and monitoring decrease in SBFI fluorescence over time. Data presented as median and error bars represent ± SEM of six individual wells from a representative experiment. **l)** RNA sequencing for mRNA levels of SLC8A2 reveals transcriptional upregulation upon treatment with FAC in *sgFth1* cells. Data represents the mean and error bars depict ± SD of three independent wells. **m)** Representative immunoblot *sgLuc* and *sgFth1* cells either untreated or treated with 100uM of FAC for 48hrs and probed for SLC8A2 and ERK II. **n)** Representative immunoblot for *sgFth1* cells infected with *Atp1a1* cDNA (ATP1a1 OE) and probed for ATP1A1 and ERK II. Statistical analysis was performed using GraphPad Prism. Unpaired, two tailed Student's t-tests were performed when comparing two groups to each other. **o)** Representative immunoblot of *sgLuc* and *sgFth1* cells infected with either empty vector (EV) or sgIL36G and either untreated or treated with FAC for 48hrs. Lysates were probed for IL36G and ERK II. **p)** Immunohistochemistry for representative images for $CD8a^+$ in liver metastases from *sgLuc* or *sgFth1* cells expressing either EV or sgIL36G. The loss of IL36G prevents $CD8a^+$ T-cell infiltration in *sgFth1* liver metastases.

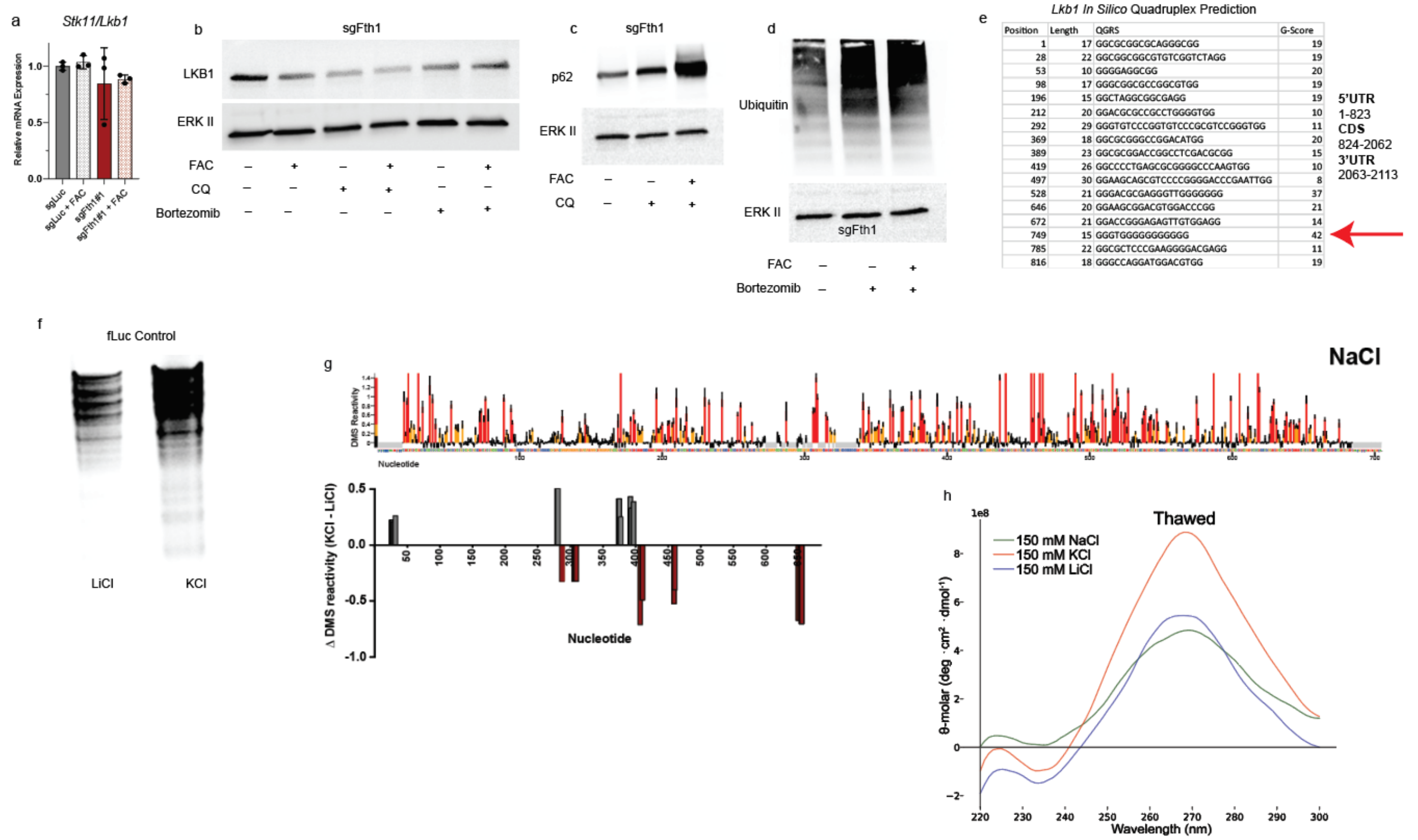

Supplemental Fig. 5 Identification of RNA G-Quadruplex in Lkb1

a) RNA sequencing for mRNA levels of Stk11/Lkb1 reveals no change in abundance upon treatment with FAC in *sgLuc* or *sgFth1* cells. Data represents the mean and error bars depict ± SD of three independent wells. **b)** Representative immunoblot of *sgFth1* cells either untreated or treated with 100uM of FAC for 48hrs. The cells were then subject to treatment with CQ (lysosomal inhibitor, 20uM) or bortezomib (proteasomal inhibitor, 100nm) for 5 hrs and probed for LKB1 and ERK II. **c)** Representative immunoblot of *sgFth1* cells either untreated or treated with 100uM of FAC for 48hrs. The cells were then subject to treatment with CQ (lysosomal inhibitor, 20uM) 5 hrs and probed for p62 and ERK II to demonstrate CQ activity. **d)** Representative immunoblot of *sgFth1* cells either untreated or treated with 100uM of FAC for 48hrs. The cells were then subject to treatment with bortezomib (proteasomal inhibitor, 100nM) 5 hrs and probed ubiquitin and Erk II to demonstrate bortezomib activity. **e)** Putative mRNA secondary structures found in Lkb1 using QGRS mapper. The higher the G-score the more likely that the sequence will form secondary structure. The 5'UTR in Lkb1 consists of base pairs 1-823. The coding sequence consists of base pairs 824-2062. The 3'UTR consists of base pairs 2063-2113. The red arrow highlights a sequence likely to form a secondary structure in the 5'UTR with a G-score of 42. **f)** Control Firefly Luciferase (fLuc) DNA was *in vitro* transcribed with T7 polymerase to make mRNA. Primers designed to the 3' end of LKB1 5'UTR were used for foot-printing the mRNA in either LiCl (control) or KCl (secondary structure inducing) conditions. Running the cDNA from RT foot-printing on a denaturing gel reveals there was no RT inhibition indicating that fLuc does not contain any mRNA secondary structures that are stabilized in the presence of KCl. **g)** Reactivity profile and secondary structure of LKB1 5'UTR refolded in the presence of NaCl. Full plot showing change in reactivity of nucleotides when refolded in the presence of KCl vs LiCl. **h)** CD spectra for thawed RNA in the presence of 150 mM NaCl, KCl, *or* LiCl. CD profiles show positive peaks at ~265 nm that are characteristic of G4 formation and are most emphasized in the KCl conditions.

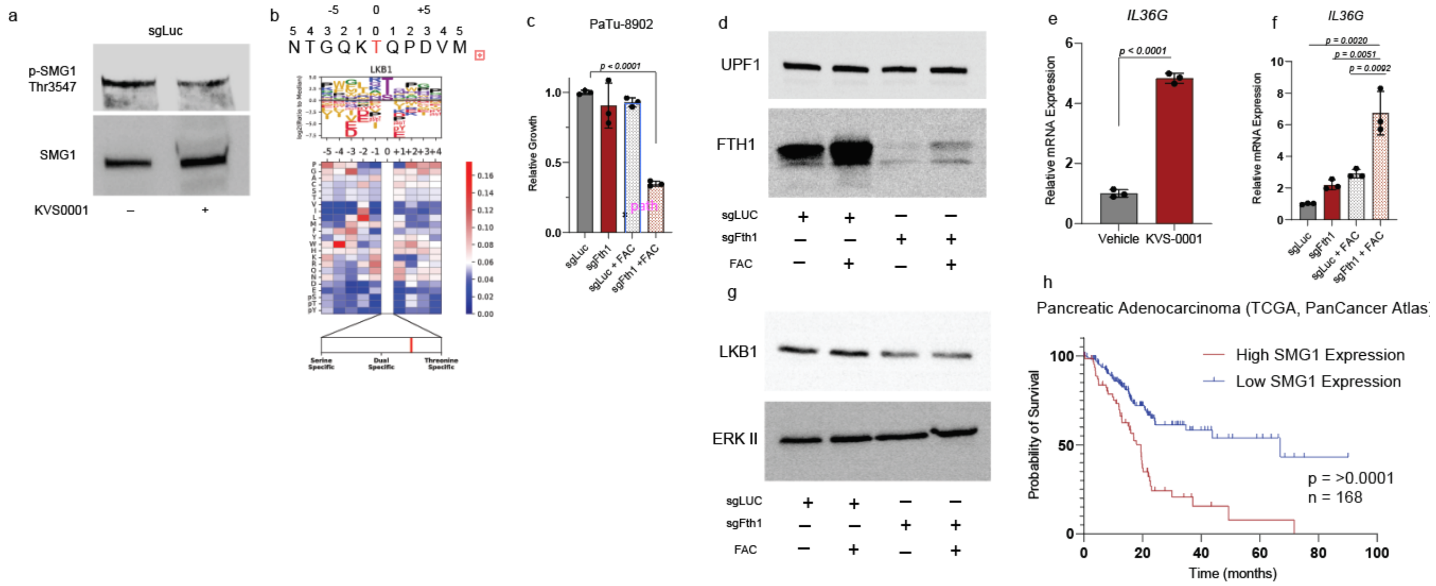

Supplemental Fig. 6 NMD perturbations and Validation in Human PDA line

a) Representative immunoblot of *sgLuc* cells that were treated with vehicle (DMSO) or KVS0001 (0.5uM) for 24hrs and probed for phospho-SMG1 Thr3547 and SMG1. **b)** Phosphosite Plus prediction of LKB1 motif at Threonine 3550 in human SMG1 (corresponds to mouse SMG1 Thr3547).[42] The top candidate kinase to phosphorylate this region of SMG1 is LKB1. **c)** Relative proliferation rates of PaTu-8902 cells infected with either sgLuc and sgFth1 cells in untreated or FAC treated conditions (100uM FAC). Data are plotted as relative cell proliferation in arbitrary units. Data presented as mean and error bars depict ± SD of three independent wells from a representative experiment. **d)** Representative immunoblot of sgLuc and sgFth1 cells either untreated or treated with FAC for 48hrs and probed for UPF1 and FTH1. **e)** RT-qPCR for IL36G in PaTu-8902 cells infected with either sgLuc or sgFth1 cells either untreated or treated with FAC for 48hrs. Data presented as mean and error bars depict ± SD of three technical replicates from a representative experiment. **f)** RT-qPCR for IL36G in PaTu-8902 cells treated with NMD inhibitor KVS-0001 for 48hrs. Data presented as mean and error bars depict ± SD of three technical replicates from a representative experiment. **g)** Representative immunoblot of sgLuc and sgFth1 cells either untreated or treated with FAC for 48hrs and probed for LKB1 and ERK II. h) Survival for pancreatic adenocarcinoma from the TCGA PanCancer Atlas. The expression of SMG1, a critical NMD kinase, is correlated with survival data of pancreatic cancer patients (p = >0.0001, n=168).

a

Position	Length	QGRS	G-Score
15	22	GGCGGCGGCGTGTCGGGCGCGG	19
39	11	GGGGGAGGCGG	21
55	24	GGCGCCCGCGAGTGAGGCGCGGGG	8
80	23	GGCGAAGGGAGCGCGGGTGGCGG	21
116	18	GGCCTTGGATGGCTGGG	20
167	17	GGCGGCCGCGGCGAGGG	19
197	29	GGGGCCCGGCACCTTCGGGAACCCCCCGG	19
312	29	GGGCACTCGGGGCGGGGCGCGCGCTCGGG	36
352	22	GGGGAGGGGGGCGCCTGCCGGG	34
392	23	GGGTCGCGGGCCGGCTCGGGGGG	35
472	23	GGCCGCGGCGGGAGGCCTCCCGG	19
524	16	GGCCGGCGGGAGTCGG	19
548	25	GGGAGGTCCGCTCGGTCGTCCGCGG	15
589	20	GGGACAGGCGGTGGGACCGG	21
639	30	GGGCTCTCCAGGTGTGGGGGTCCCGGGGGG	30
694	16	GGGATGGGCGGCCCGG	20
747	19	GGCCTGAGGTCCCCGGAGG	17
789	26	GGCCGGCCTCCCCAGGTCCCCGAGG	15
980	30	GGAAGAAGGGTTTTTCCCTTCCTTTTGGGG	5
1052	25	GGAGAAGGGAAGTCGGAACACAAGG	19
1102	12	GGCTGGCGGCGG	20

Putative G-Quadruplex

b

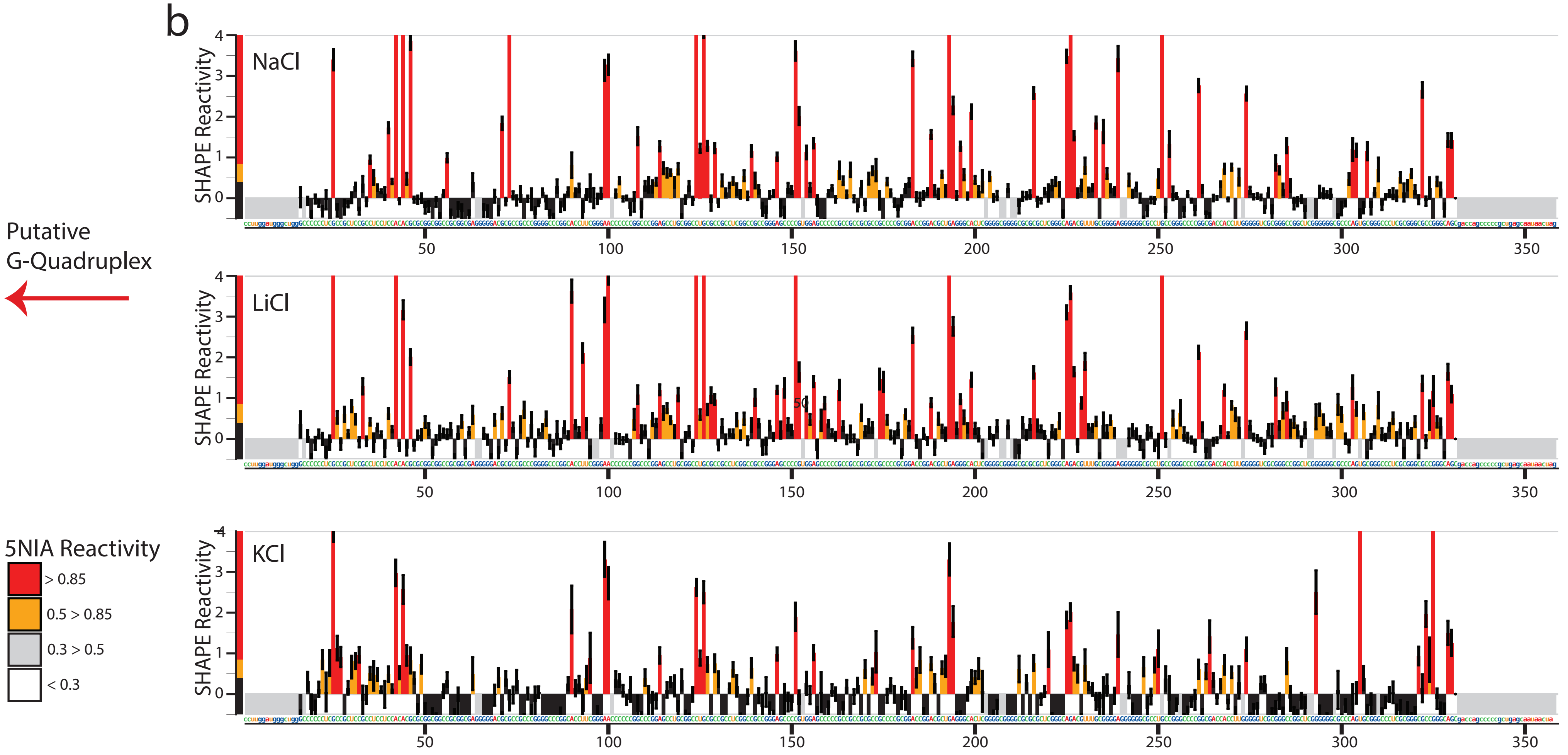

c

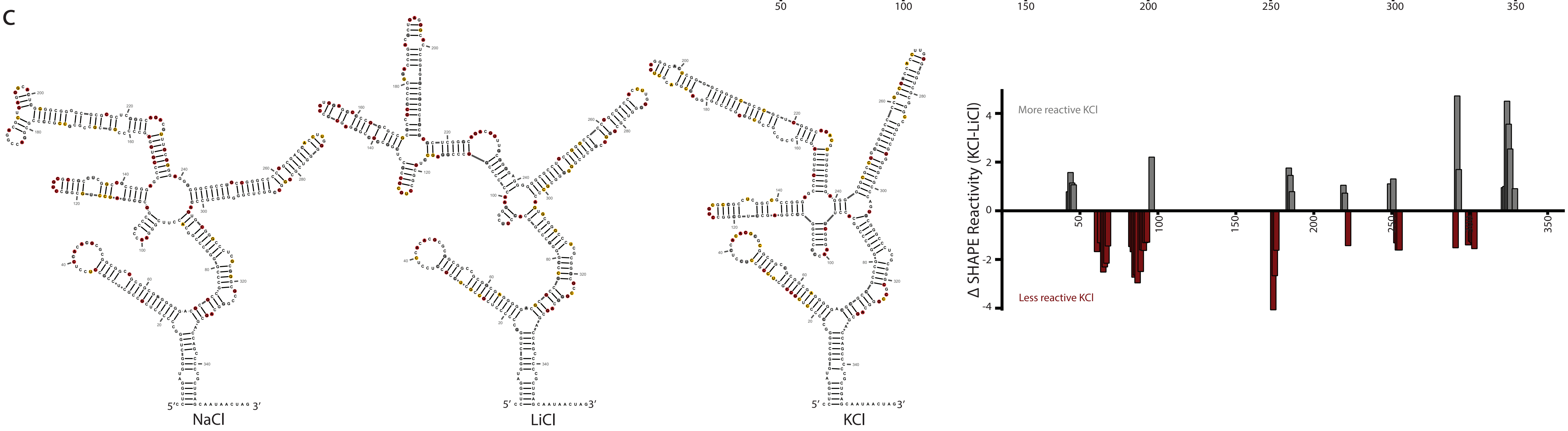

Supplemental Fig. 7 Identification of RNA g-quadruplex in Human Lkb1
a) Putative mRNA secondary structures found in human LKB1 using QGRS mapper. The higher the G-score the more likely that the sequence will form secondary structure. The 5’UTR in LKB1 consists of base pairs 1-1136. The coding sequence consists of base pairs 1137-2438. The 3’UTR consists of base pairs 2439-3293. The red arrow highlights a sequence likely to form a secondary structure in the 5’UTR with a G-score of 36. **b)** Reactivity profile and secondary structure of human LKB1 5'UTR refolded in the presence of NaCl. Full plot showing change in reactivity of nucleotides when refolded in the presence of KCl vs LiCl **c)** Human LKB1 5’UTR secondary structures determined via chemical probing.

DataS1: Liver Metastasis Screen and RNA Sequencing Data

This table reports gene-level analysis of sgRNA depletion in liver metastases, subcutaneous tumors in C57BL/6 mice, analysis of differential sgRNA depletion in liver metastases compared to subcutaneous tumors in C57BL/6 mice, RNAseq data for FTH1 control and KO cells treated with FAC, and RNAseq data for FTH1 control and KO cells.
